# Saccadic Suppression of Displacement Does Not Reflect a Saccade-Specific Bias to Assume Stability

**DOI:** 10.3390/vision3040049

**Published:** 2019-09-24

**Authors:** Sabine Born

**Affiliations:** Faculté de Psychologie et des Sciences de l’Education, Université de Genève, 1205 Geneva, Switzerland; sabine.born@unige.ch; Tel.: +41-22-379-9122

**Keywords:** saccades, masking, displacement perception, spatial stability, motion perception

## Abstract

Across saccades, small displacements of a visual target are harder to detect and their directions more difficult to discriminate than during steady fixation. Prominent theories of this effect, known as saccadic suppression of displacement, propose that it is due to a bias to assume object stability across saccades. Recent studies comparing the saccadic effect to masking effects suggest that suppression of displacement is not saccade-specific. Further evidence for this account is presented from two experiments where participants judged the size of displacements on a continuous scale in saccade and mask conditions, with and without blanking. Saccades and masks both reduced the proportion of correctly perceived displacements and increased the proportion of missed displacements. Blanking improved performance in both conditions by reducing the proportion of missed displacements. Thus, if suppression of displacement reflects a bias for stability, it is not a saccade-specific bias, but a more general stability assumption revealed under conditions of impoverished vision. Specifically, I discuss the potentially decisive role of motion or other transient signals for displacement perception. Without transients or motion, the quality of relative position signals is poor, and saccadic and mask-induced suppression of displacement reflects performance when the decision has to be made on these signals alone. Blanking may improve those position signals by providing a transient onset or a longer time to encode the pre-saccadic target position.

## 1. Introduction

### 1.1. Saccadic Suppression of Displacement and Visual Stability across Eye Movements

In our everyday experience, we have the impression that no important change in our environment escapes us. Contrary to this impression, it has been demonstrated that even large changes of the visual scene can go unnoticed if the change is not accompanied by a visible and localizable transient that draws attention towards it [1]. For instance, the change might be camouflaged when it occurs simultaneously with a saccadic eye movement. In particular, small displacements (‘jumps’) of a visual object that are easily seen during fixation may be missed when they happen during a saccade, even when the object was the target of the eye movement [2,3,4,5,6,7]. This effect is known as saccadic suppression of displacement. It is thought to be intrinsically linked to the problem of visual stability across eye movements. There are several facets to this problem [8]. Most relevant to saccadic suppression of displacement is the question how we can keep track of an object’s location across saccades, despite the fact that each eye movement we make results in drastic shifts of the visual image projected onto our retina. It has long been assumed that the visual system integrates extraretinal information about the size of the saccade (e.g., efference copy or proprioceptive eye position signals) with the sensory information about the object’s position, see [9] for a historical perspective. However, if we have such a mechanism, why are small displacements nonetheless more difficult to detect or discriminate across saccades than during fixation?

### 1.2. The Blanking Effect

Probably the simplest explanation is that the addition of the extraretinal signal adds further noise compared to a situation without concurrent eye movement. In a simple signal/noise ratio model, this alone could explain the increase in displacement detection thresholds [5,10]. This simple model has been challenged, however, by the observation that displacement perception is strongly improved when the target is removed during the saccade, reappearing in its displaced position only after the saccade has landed [6,11,12,13,14,15,16]. This so-called blanking effect described by Deubel, Schneider, and Bridgeman in 1996 [6] has been a seminal finding in saccadic suppression of displacement. In the current contribution, I will therefore focus on studies that have been published in or after 1996, see [8] and related commentary for discussion of earlier work. The blanking effect suggests that location information across saccades is more precise than initially presumed. There is now evidence that the available information may even include the trial-by-trial saccadic motor error, that is, the difference between the planned and the actual saccade size [17,18]. Thus, theories of saccadic suppression of displacement do not only have to explain why displacement perception is worse during saccades than during fixation. Maybe more importantly, they also have to explain the counter-intuitive blanking effect; that is, why performance is better when the target is absent when the eyes land. 

### 1.3. Saccadic Suppression of Displacement as the Result of a Bias for Stability

One prominent line of argument today posits that saccadic suppression of displacement reflects a perceptual decision bias to assume stability across saccades [6,7,11,12,13]. Based on an object’s pre-saccadic location and extraretinal signals of the size of the saccade, a prediction of the object’s post-saccadic location may well be formed. In the case of the saccade target, the prediction does not even need to incorporate extraretinal signals, as the target should always be located close to the fovea after the eyes have landed [6,7,19]. The prediction could then be compared to the object’s incoming visual signal after the saccade. However, it is assumed that this information is often not used, as long as the target was found near the predicted location after the saccade. Unless the discrepancy between prediction and incoming signal is very large (as is the case for larger jumps), the visual system per default assumes the null hypothesis that no displacement has occurred [6,7,11,12,13]. Alternatively, Niemeier and colleagues [16,20] have proposed a prior for stability that biases the perceived size of displacements. The specific shape of the prior leads to underestimations of jump size over a range of smaller displacements. For the smallest displacements, the prior leads to almost complete superposition of the pre- and post-saccadic signals (‘contraction’). Consequently, the smallest jumps remain undetected.

The idea of a perceptual null hypothesis has already been expressed earlier, for instance by MacKay [21] as cited in [8]. The blanking effect, however, has helped the idea of a decision bias or prior for stability to achieve widespread acceptance. To explain the blanking effect, it has been proposed that the stability bias can be relaxed, when there is evidence suggesting a break in object continuity or object correspondence across the saccade: for instance, if the target is not present upon saccade landing, as in the blanking effect [6,11,12,13,14,15,16]. In other words, if the visual system has already one clear indication that the object has undergone a change, signals that suggest a concurrent change in position are more easily trusted or receive more weight in the estimation of the displacement.

### 1.4. Is the Stability Bias Saccade-Specific?

Although the proposed bias may not only be effective across eye movements, it is quite remarkable that suppression of displacement is almost exclusively discussed in the context of saccades. The specifications ‘saccadic’, ‘across saccades’ or similar terms are found in the titles of almost all studies [6,7,11,12,13,14,15,16,22,23,24,25,26]. Moreover, the specific challenge of spatial updating across saccades, that is, having to assess the displacement across a large shift of the entire retinal image brought on by the eye movement, is mentioned in most introductory passages [7,11,12,13,14,15,16,19,20,22,23,24,25,26]. Due to this emphasis on the saccadic context, one may be tempted to assume that the proposed mechanisms are saccade-specific, and closely related to the saccade-specific challenge of spatial updating. Taking the idea of a null hypothesis of stability as an example, one may be tempted to assume that the saccadic context raises the decision threshold for displacement perception: A difference in an object’s position signal may be interpreted as a displacement during fixation, but a same-sized difference between the predicted location of the object and its actual post-saccadic signal may not be sufficient to reject the perceptual null hypothesis in the context of a saccade. However, many saccadic suppression of displacement studies did not compare performance to any kind of non-saccadic control condition [11,12,13,14,15,20,22,23,24,25,26]. It remains thus unclear whether similar mechanisms may operate during fixation and whether estimations across eye movements are more prone to the suggested stability bias or not.

### 1.5. Saccadic Suppression of Displacement as the Result of Saccadic Suppression of Vision

It is important to note that estimations across saccades are not only difficult because of the large shift of the retinal image. It is well established that saccades have detrimental effects on the visual input stream, e.g., [27,28,29]. These effects have been termed saccadic suppression of vision or visibility and have been largely examined separate from saccadic suppression of displacement, as they have been linked more closely to another facet of visual stability across saccades (namely why we do not perceive motion blur, that is, the entire scene sweeping across the retina, when we make saccades). Nevertheless, vision is of course also impoverished during situations of saccadic suppression of displacement. Thus, saccadic suppression of displacement may primarily reflect poor vision during saccades. To assess how much of saccadic suppression of displacement can be explained by poor vision and how much may be specific to the challenge of spatial updating, a fixation control condition with similarly impoverished vision of the target jump may be helpful.

Indeed, recent experiments have reported difficulties in discriminating small jumps of a peripheral target in a situation without saccades, when the jump was obscured by a full-screen pattern mask [30,31]. The effects were of similar magnitude as the saccadic effect. Further, prolonging the preview duration of the target before the displacement led to similar improvements with masks and saccades [31] and an improvement with blanking was also found in both conditions [30]. When it comes to masking, the general take on observed performance decrements is not that they primarily reflect a bias, but that they reflect the reduced visibility of the target [32]. Similarly, rather than highlighting a stability bias, saccadic suppression of displacement may primarily reflect the saccade’s masking effect on the visual input stream [30]. Specifically, the masking of transient onset or motion signals (usually perceived when the target jumps) may play a crucial role. In this framework, saccadic suppression of displacement reflects poor sensitivity due to missing clues, not a bias to assume stability. That is, position signals alone are coarse to begin with, even without saccades, and especially when the time available for their encoding is short [31]. Consequently, when having to rely on position signals alone in saccade or mask conditions, displacement perception is poor [33]. In turn, the improvement with blanking may not reflect a relaxed bias, but could be due to better visual evidence for a displacement, for instance, by providing a clear onset transient when the target reappears [6,25,31], or by providing more time for the pre-jump position to be encoded [31].

### 1.6. Do Saccades and Masks Affect Displacement Perception in Similar Ways?

In accordance with many saccadic suppression of displacement studies, displacement direction discrimination tasks were used in previous masking experiments [30,31]. Participants had to decide on each trial, whether the target had jumped forward (i.e., further out into the periphery) or backward (i.e., towards fixation). Responses are hard to interpret in these tasks: If participants gave a false response, did they truly see a jump opposite to the actual displacement, or did they just guess because they did not see a displacement at all? Could it be that a higher decision threshold is applied during saccades, whereas poor performance in masking conditions rather reflect more noise in the signal compared to the control condition without mask? In the current experiments, I therefore asked participants to estimate the size of the displacement by indicating the target’s starting position through a mouse click. Similar continuous measures have already been used to measure suppression of displacement, but only in saccade conditions [16,20,26]. Continuous report techniques may provide more information about the processes underlying the responses. For instance, in the literature on visual working memory, continuous report techniques have successfully provided tools to quantify the probability and fidelity of memory representations [34,35]. Similarly, a continuous report technique may be better suited to assess the probability and fidelity of perceiving a displacement in control, saccade and mask conditions.

## 2. Experiment 1: Displacement Estimates in Control, Saccade and Masking Conditions

**Figure 1:**
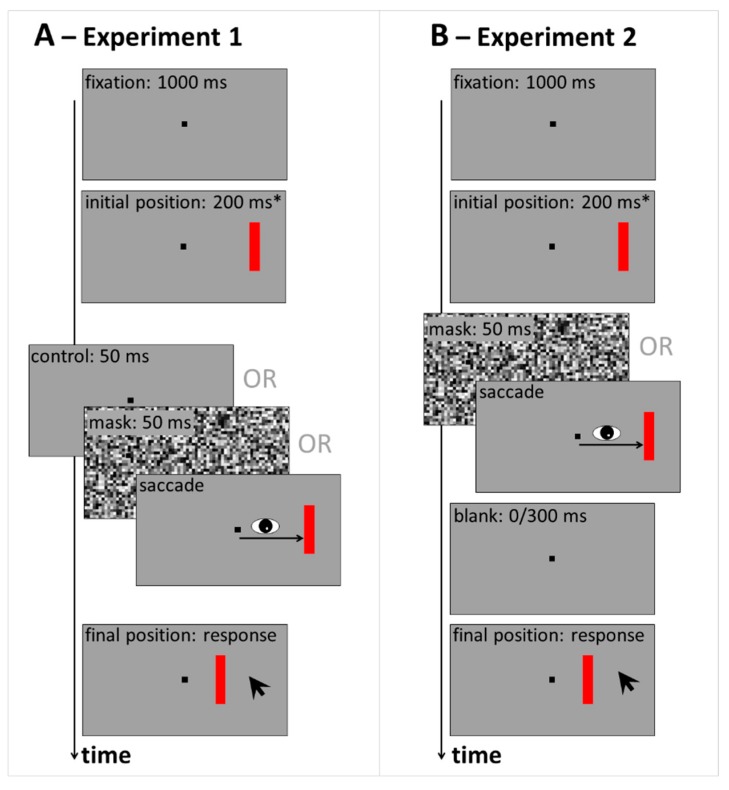
Sequence of events in Experiment 1 (**A**) and Experiment 2 (**B**). Participants fixated the central black square throughout a trial, or made a saccadic eye movement towards the red target bar. The target was displaced either after 200 ms, * or as soon as the saccade was detected. At the end of each trial, participants indicated the perceived displacement size by clicking on the target’s initial (before displacement) position on the screen.

Experiment 1 compares response distributions across control, saccade and mask conditions. The procedure in Experiment 1 is illustrated in Figure 1A. Details on the methods can be found after the General Discussion. Demo movies of the control (Video S1) and mask (Video S2) conditions can be found in Supplementary Online Material.

### 2.1. Direction Discrimination: Suppression of Displacement (Recoded Data)

**Figure 2:**
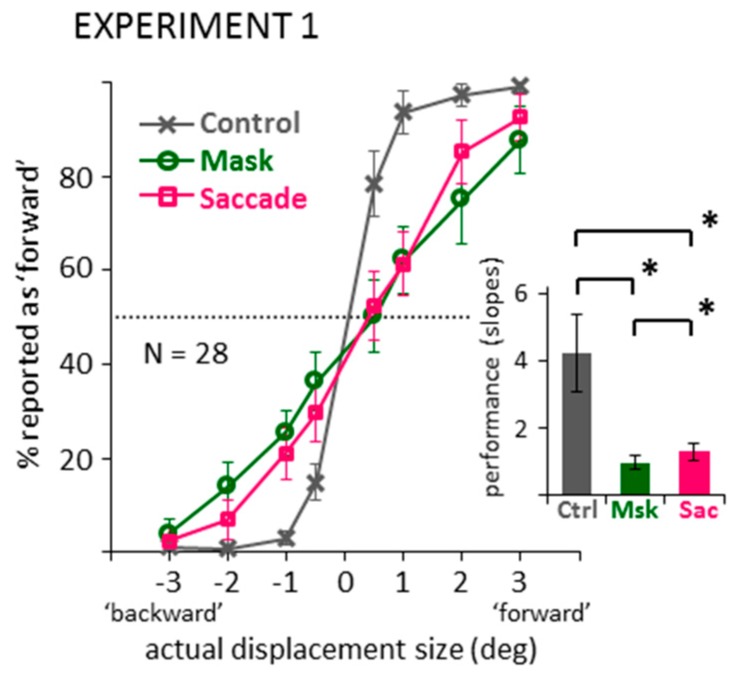
Data from the control, mask and saccade conditions of Experiment 1, recoded into binary displacement direction responses. Line graph shows actual displacements (negative: ‘backward’ vs. positive: ‘forward’) against the percentage of reported ‘forward’ responses (dotted line: chance level). Bar graph shows the average slopes of the logistic functions fitted to each participant’s individual data. All error bars: between-subjects 95% confidence intervals. Brackets marked with * illustrate significant differences in post-hoc paired-samples *t*-tests (*p* < 0.05).

First, to check whether changing the response mode from direction discrimination to mouse pointing gives results that are comparable to previous suppression of displacement experiments, data were first recoded into binary responses, that is, into reported ‘forward’ and ‘backward’ displacements. Figure 2 illustrates the percentage of trials recoded as a reported ‘forward’ displacement for the two jump directions (‘backward’ or ‘forward’ as indicated by negative or positive numbers, respectively) and four jump sizes (0.5, 1, 2, 3 deg) in the three conditions (control, mask, saccade). The slope of the data pattern can be taken as an estimate of performance: The steeper the curve, the better participants’ discrimination of jump direction. To summarize performance, logistic functions were fit to the data of each individual observer in the three conditions, using a maximum-likelihood method in Matlab (see https://osf.io/atr6y/). Average slopes of these functions are illustrated in the bar graph of Figure 2. A one-way ANOVA on these slope values revealed a significant main effect, *F*(2,54) = 30.56, *p* < 0.001, partial *η^2^* = 0.531, and subsequent paired-samples *t*-tests and Bayesian *t*-tests (default Bayesian *t*-tests as offered in JASP [36,37]) confirmed significantly shallower slopes in the mask and saccade compared to the control condition, *t*s(27) > 5.52, *p*s < 0.001, *BF*_10_ > 2762.75. Thus, by recoding the data, the classic suppression of displacement effect emerged: We see impaired performance in direction discrimination in the saccade, but also the masking condition, compared to control. The slopes in the mask and saccade condition also showed a significant difference, *t*(27) = 2.67, *p* = 0.013, *BF*_10_ = 3.78, indicating a slightly shallower slope in the mask compared to the saccade conditions. However, this difference was much smaller compared to the large differences to the control condition.

### 2.2. Distributions of Displacement Estimates

**Figure 3:**
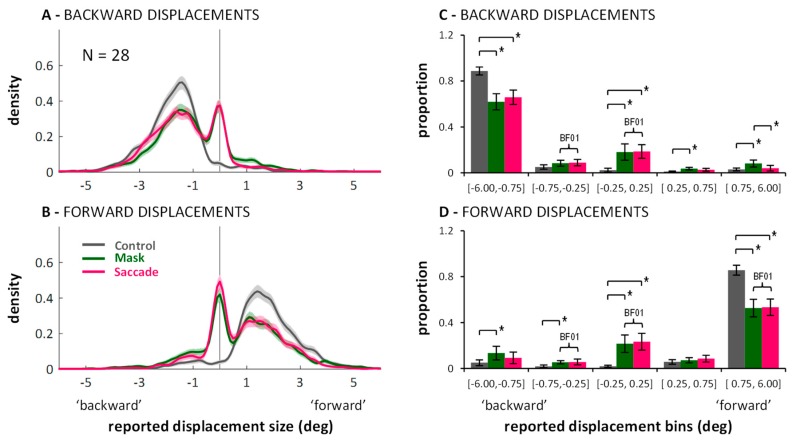
Distribution of responses in the three conditions (color-coded) of Experiment 1: Kernel density functions showing data combined across participants (panels (**A**,**B**); shadings represent bootstrapped 95% confidence intervals), and proportion of responses falling into the specified bins showing data averaged across participants (panels (**C**,**D**); error bars represent between-subjects 95% confidence intervals). Brackets marked with * illustrate significant differences in post-hoc within-subjects paired t-tests, *p* < 0.0017). Curly braces marked with BF01 illustrate Bayes factors in favor of H0, *BF*_01_ > 3.00. Data are shown separately for backward (upper row) and forward (lower row) displacements.

**Figure A1:**
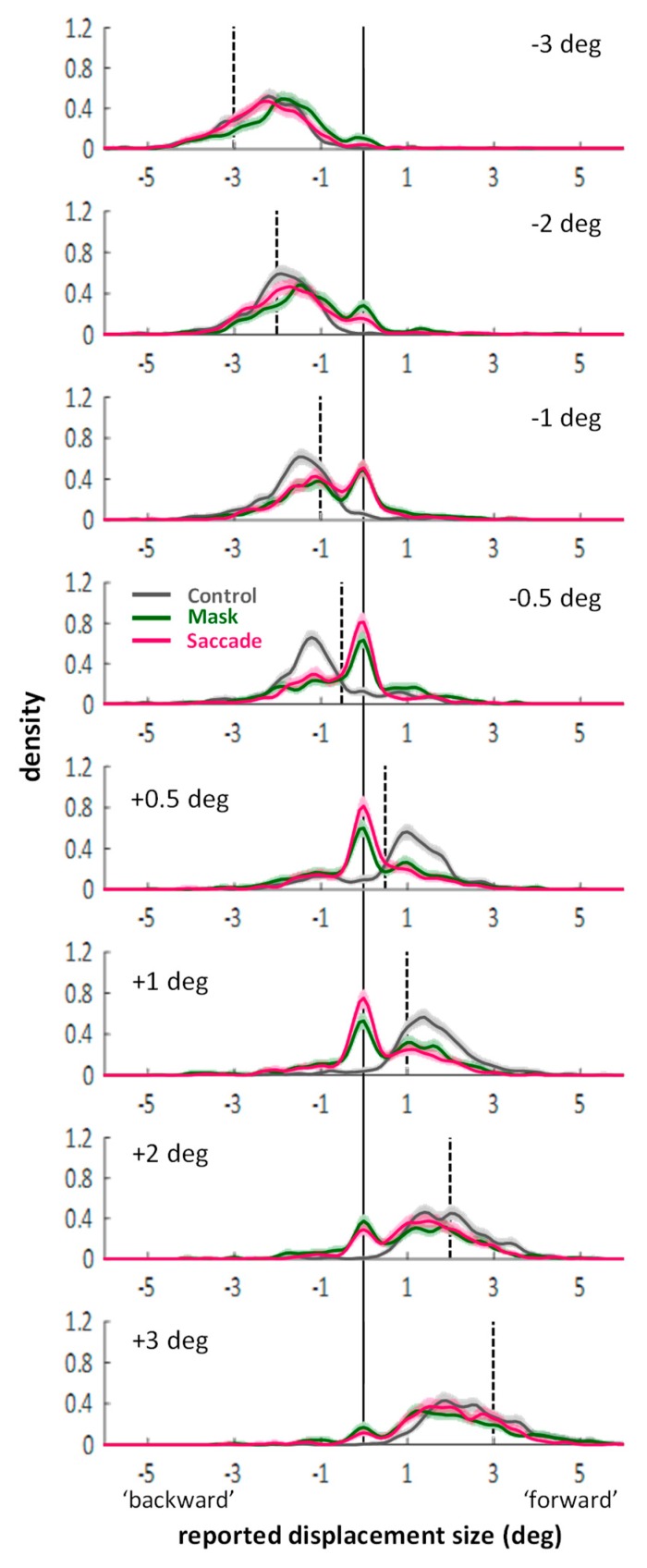
Distribution of responses (kernel density functions) in the three conditions of Experiment 1 (color-coded). Rows represent the different displacement sizes tested. Data are collapsed across all 28 participants. Shadings represent bootstrapped 95% confidence intervals. Vertical black continuous line: zero line. Vertical black dashed lines: actual displacement for each graph. Compared to actual displacements, the larger ±2 and ±3 deg displacements were underestimated in all conditions (distributions shifted towards zero compared to the dashed lines). The smaller ±0.5 and ±1 deg displacements, however, were overestimated in the control condition. Missed displacements, i.e., peaks around zero emerged for all displacements in the saccade and mask conditions and became more frequent with smaller displacements. All in all, distributions in mask and saccade conditions resembled each other, while distributions in the control conditions were markedly different for smaller displacements as virtually no misses occurred.

Next, I visualized the distributions of the continuous mouse click responses. Figure A1 in Appendix A shows the distributions separate for each displacement size. The essential aspect, however, can also be appreciated in Figure 3 which shows the response distributions separated only by backward (Figure 3A) and forward (Figure 3B) displacements. For the density functions, a kernel smoothing technique based on a Gaussian kernel function (ksdensity in Matlab) was applied to the combined data of all twenty-eight participants (kernel bandwidth: 0.15 deg). The shaded areas around each line represent bootstrapped 95% confidence intervals (based on 1000 samples with replacement). Distributions in the control condition (grey lines) are unimodal and responses around zero (i.e., misses) or false responses (i.e., on the “wrong” side of the vertical line marking zero displacement) are rare. In contrast, the curves in the saccade (magenta) and mask (green) conditions are clearly bimodal. There is always one peak around zero, subtending approximately ±0.5 deg, with flanks falling off symmetrically on both sides. The remaining part of the distributions fall under the one from the control condition. The two peaks are separated by troughs, which suggests that the peak around zero represents the proportion of trials on which participants completely missed the displacement, whereas the second peak represents the proportion of trials on which a displacement was perceived correctly as a backward or forward displacement. (Figure A1 in Appendix A shows that the location of the non-zero peak roughly follows the actual displacement size.) The graphs suggest that suppression of displacement (Figure 2) for the saccade and mask conditions may to a large part be explained by higher proportions of missed displacements compared to the control condition, as evident in the marked peaks around zero. (Figure A1 in Appendix A also shows that the size of the peaks around zero scale with displacement size: smaller displacements are more often missed. However, missed displacements did not only occur for the smallest displacements.) 

To see whether the observed pattern is robust across participants, I split each participant’s responses in the three conditions into five bins, defined post-hoc on the basis of the kernel density function: The three central bins have the same size: [−0.75, −0.25], [−0.25, 0.25], and [0.25, 0.75], representing missed displacements and the two adjacent bins where responses are ambiguous; the two outer bins are larger [−6.00, −0.75] and [0.75, 6.00], representing the trials on which participants clearly indicated a displacement, either in the correct direction, or in the wrong direction. The data (illustrated in Figure 3C,D) was entered into a 2 (displacement direction: backward or forward) × 3 (condition: control, saccade, mask) × 5 (bins) repeated measures ANOVA. Importantly, the three-way interaction was significant, *F*(8,216) = 58.08, *p* < 0.001, partial *η*^2^ = 0.683. To examine how the different conditions changed the distribution of responses across bins, post-hoc paired *t*-tests were conducted, with *p*-level corrected to 0.05/30 = 0.0017. Significant differences on this level are highlighted in Figure 3C,D with brackets and an asterisk. These reveal that in saccade and mask conditions, the proportion of correctly reported displacements (i.e., >0.75 deg and in the right direction) diminished compared to control, whereas the proportion of missed displacements in the range [−0.25, 0.25] increased significantly. While these comparisons cannot attest that distributions were bimodal for all participants, importantly, they do affirm that the peak around zero as found in the kernel density functions does not result from only a small subset of participants. No differences across conditions were found in the bin in-between, corresponding to the trough in the kernel density function (i.e., [−0.75, −0.25] for backward, and [0.25, 0.75] for forward displacements). Additionally, masking, but not saccades, led to higher proportions of displacement reports in the wrong direction. Figure 3C,D also highlights which comparisons resulted in Bayes factors in favor of H0 larger than *BF*_01_ > 3.00 (again: default Bayesian *t*-tests as offered in JASP [36,37]). Those include the comparison between saccade and mask conditions for the central [−0.25, 0.25] bin for both forward and backward displacements.

To summarize, saccades and masks modulated the response distributions in quite similar ways. Both primarily seemed to increase the proportion of missed displacements. When taken in isolation, the bimodality in the distributions in the saccade condition can be easily explained by accounts that emphasize a stability bias across saccades [6,7,11,12,13,16]. A similar distribution was observed in the masking condition, however. Thus, the bias does not seem to be contingent on the saccadic situation.

## 3. Experiment 2: Estimates of Displacement Size in Blanking Conditions with Saccades or Mask

If saccades result in a larger proportion of missed trials, how does a blank introduced during a saccade produce a performance increase, as has been reported in many previous studies [6,11,12,13,14,15,30]? One prominent explanation for the blanking effect in saccades posits that the absence of the target upon saccade landing signals a break in object continuity e.g., [6,11,13,16]. As the visual system has already one clear indication that the object has undergone a change, the strong bias towards spatial stability is relaxed. Consequently, the proportion of reported misses should decrease. Given that the mask condition produced a similar pattern of responses in Experiment 1, the same reduction of misses may also be observed with masking. Further, one may wonder whether blanking has only positive effects on displacement perception. It has been argued that a bias towards stability across saccades could optimize perception by correctly discarding small mismatches between prediction and actual post-saccadic position. In particular, small deviations between the extraretinal signals and the actual saccade should result in the perception of small illusory jumps of the visual scene with almost every saccade we make, as discussed e.g., in [8,12,19]. If the bias is relaxed, then this additional noise should show through more often. In other words, the relaxed decision threshold should also lead to a slightly higher proportion of trials in which participants report the wrong jump direction. Indeed, it has been found that blanking conditions increase the false alarm rate in displacement detection tasks [24]. To examine these issues, Experiment 2 compares blank and no-blank conditions in saccade, but also mask conditions (see Figure 1B). A demo movie of the 300 ms blank with mask condition (Video S3) can be found in Supplementary Online Material.

### 3.1. Direction Discrimination: Suppression of Displacement (Recoded Data)

**Figure 4:**
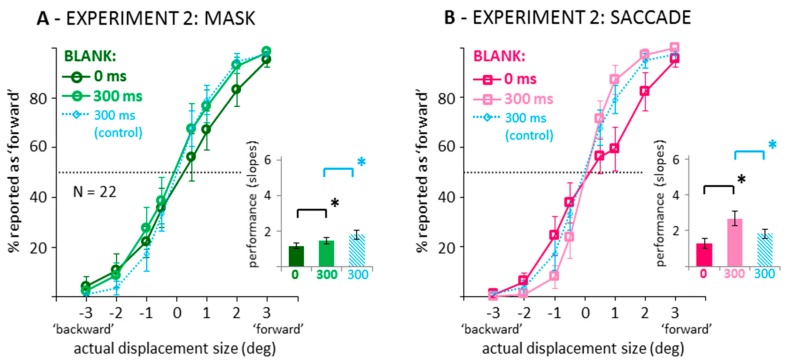
Data from the 0 and 300 ms blank conditions in the mask (**A**) and saccade (**B**) conditions of Experiment 2, recoded into binary displacement direction responses. Conventions as in Figure 2. Thin blue lines and striped blue bars represent data from an additional experiment (see Appendix B) with a 300 ms blank during fixation condition without mask.

Figure 4 illustrates the results when recoding the data into binary responses (backward/forward displacement direction discrimination). In saccade and mask conditions, curves are steeper, that is, discrimination performance increased when introducing a 300 ms blank after the saccade or mask. The slope difference between the 0 and 300 ms blank conditions was larger in the saccade condition. Figure 4A suggests that in the masking condition, facilitation by blanking only occurred for forward, but not for backward displacements. A two-way ANOVA on the corresponding slopes (see bar graphs in Figure 4) confirmed significant main effects of blanking, *F*(1,21) = 75.22, *p* < 0.001, partial *η*^2^ = 0.782, and condition, *F*(1,21) = 20.65, *p* < 0.001, partial *η*^2^ = 0.496, and a significant interaction, *F*(1,21) = 29.62, *p* < 0.001, partial *η*^2^ = 0.585. Although smaller in the masking condition, subsequent paired-samples *t*-tests confirmed a blanking effect, that is, a significantly steeper slope in the blanking condition in both the saccade as well as the mask conditions, *t*s(21) > 4.37, *p*s < 0.001, *BF*_10_ > 114.20. Also, Figure 4 shows data from an additional Experiment 3 in which a fixation condition without mask was tested with a 300 ms blank across the displacement (see Appendix B for the full data set; Video S4 for a demo). Independent-samples *t*-tests comparing this 300 ms blank during control condition (light blue data) to the 300 ms blank during masking condition revealed a significant result, *t*(48) = 2.10, *p* = 0.041, pointing to slightly better performance without masking, although the Bayes factor suggests that evidence in favor of a difference was very weak, *BF*_10_ = 1.65. In contrast, the slopes of the psychometric functions were significantly steeper, that is, performance was better, in the 300 ms blank during saccade than the control condition from the additional experiment, *t*(48) = 3.80, *p* < 0.001, *BF*_10_ = 66.78.

### 3.2. Distributions of Displacement Estimates

**Figure 5:**
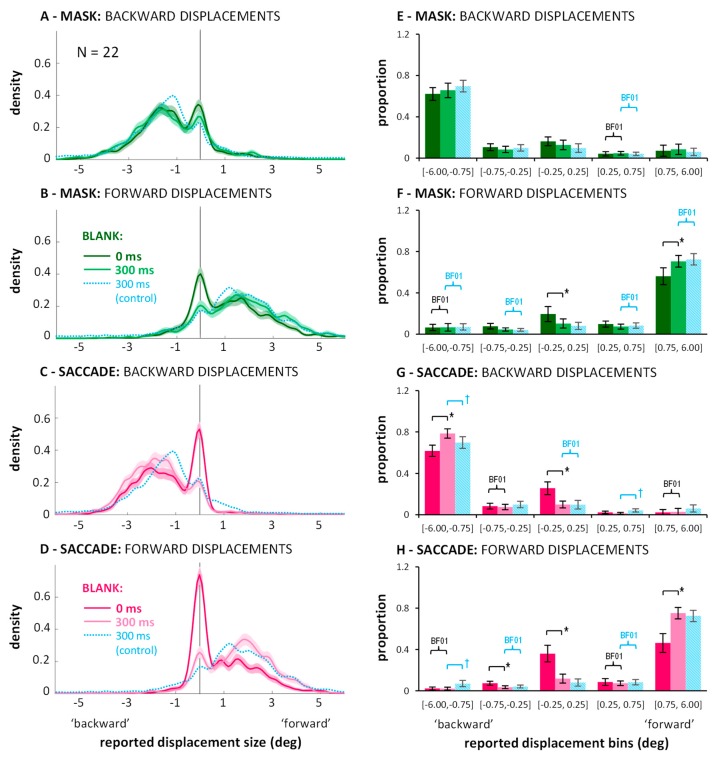
Distribution of responses in the saccade (magenta) and mask (green) conditions of Experiment 2, with (light colors) or without (saturated colors) a 300 ms blank: Kernel density functions (panels (**A**–**D**), and proportion of responses falling into the specified bins (panels (**E–H**). Conventions as in Figure 3. Thin blue lines and striped blue bars represent data from a 300 ms blank during fixation condition without mask (Appendix B). Brackets marked with * illustrate significant differences in post-hoc within-subjects paired *t*-tests (*p* < 0.0025). Brackets marked with † illustrate significant differences in post-hoc between-subjects *t*-tests (*p* < 0.05). Curly braces marked with BF01 illustrate Bayes factors in favor of H0, *BF*_01_ > 3.00.

Can the poor performance in saccade and mask conditions again be explained by a high proportion of missed displacements? Is the improvement through blanking due to a reduction in missed displacements? And does the benefit of blanking come at the expense of more false direction responses in saccade or mask conditions? Figure 5 (left column) shows the distributions of jump size estimates in each condition with data from all twenty-two participants combined. 

**Figure A2:**
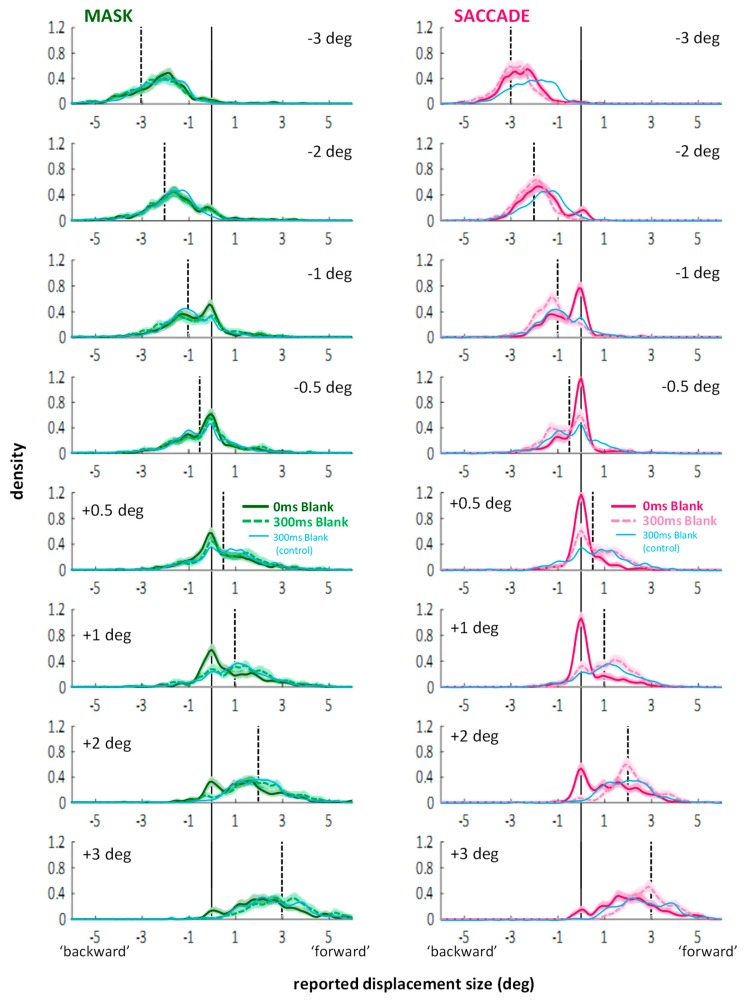
Distribution of responses (kernel density functions) in the mask (left) and saccade (right) conditions of Experiment 2. Continuous lines: no blank conditions, dashed lines: blank conditions. Thin blue lines represent data from the 300 ms blank condition during fixation from Experiment 3. Improvements with blanking (i.e., less misses) in the masking conditions were most visible in the +1 and +2 deg displacement conditions (with smaller modulations in the −1 and +0.5 deg conditions). Distributions overlap almost perfectly with the ones from the 300 ms blank conditions of Experiment 3. In the saccade conditions, less misses compared to the no blank condition occurred for all displacements (except for the −3 deg displacements where no misses occurred). Differences between the 300 ms blank conditions (Experiment 2 vs. Experiment 3) were subtle, but showed, for instance, in larger estimates in the saccade condition and an absence of false direction responses (see −0.5 deg: some displacements were judged to be around +1 deg in the blank during fixation condition of Experiment 3).

In general, the curves show again two peaks: One around zero perceived displacement, representing the proportion of missed displacements, and a second peak, representing the trials on which a displacement was perceived in the correct direction. Comparing data from the no blank (saturated colors) to the blanking conditions (lighter colors), the improvements in direction discrimination performance indeed went along with an altered balance between those two peaks: A lower proportion of missed trials and a higher proportion of perceived displacements. Entering the corresponding proportions (see Figure 5, right column) into a 2 (displacement direction: backward or forward) × 2 (condition: mask or saccade) × 2 (blank: 0 vs. 300 ms) × 5 (bins) repeated-measures ANOVA, a significant four-way interaction emerged: *F*(4,84) = 8.62, *p* < 0.001, partial *η*^2^ = 0.291. Conducting separate ANOVAs for the mask and saccade conditions still resulted in significant three-way interactions in both cases: *F*s(4,84) > 9.82, *p*s < 0.001, partial *η*^2^ > 0.319. Thus, although the modulations in the saccade condition may be larger, there were also significant modulations in the distributions of trials across the bins through blanking in the masking condition. Results of post-hoc paired samples *t*-tests are illustrated by the brackets in Figure 5 (*p*-level corrected: 0.05/20 = 0.0025). In the saccade condition, the change in balance (more correctly estimated displacements, less missed displacements) resulted in significant differences for both jump directions (see Figure 5G,H; black brackets). Further, the bin in-between correctly judged and missed displacement contained a similar proportion of trials in blank and no blank conditions as indicated by Bayes factors in favor of H0, *BF*_01_ > 3.00 (black curly braces). In the masking condition, significantly more responses in the bin containing the larger jump estimates, paired with significantly fewer responses around zero with blanking were only evident for forward displacements (see Figure 5F; black brackets). Due to small variations in the distributions, Bayes factors in favor of H0, *BF*_01_ > 3.00 (black curly braces), were revealed only for two bins not immediately relevant for the current questions. False direction responses remained overall rare. (They occurred occasionally for small displacements in the masking conditions; see: distributions for ±0.5 deg displacements in Figure A2 in Appendix A: they extend to values up to 2–3 deg to the other side, although the proportions of those false direction estimates do not seem substantially higher with blanking). In the saccade condition, however, distributions drop sharply on the ‘wrong’ side and blanking did not evoke more false direction responses.

The faint blue lines in Figure 5 and the striped blue bars represent data from the 300 ms blank during fixation condition from the additional experiment without mask (Appendix B). In the masking condition, distributions are similar to the blank distributions with masking. When entering the proportions of the two 300 ms blank conditions into a 2 (displacement direction: backward or forward) × 5 (bins) × 2 (experiment: with or without masking) mixed-measures ANOVA, neither the main effect nor any interaction with the between-subject factor experiment reached significance, *F*s < 1.65, *p*s > 0.205. Thus, one may say that for forward displacements, blanking after the mask improved performance roughly up to the level without masking. This is further highlighted by the fact that most bins show Bayes factors in favor of H0, *BF*_01_ > 3.00, for forward displacements (see blue curly braces in Figure 5F; the central bin is at *BF*_01_ = 2.57). In contrast, comparing the 300 ms blank during fixation condition to the blank with saccade condition, the corresponding ANOVA did reveal a significant three-way interaction, *F*(4,192) = 5.74, *p* < 0.001, partial *η*^2^ = 0.107. The small effect size and the post-hoc *t*-tests indicated that differences were subtle: None of the *t*-tests comparing proportions across experiments reached significance for any of the bins when correcting for multiple comparisons (*p*-level corrected: 0.05/20 = 0.0025). Figure 5G,H (see blue brackets illustrating significant tests at *p* < 0.05) suggest the three-way interaction may be partly explained by less false direction responses in the saccade compared to the blanking during fixation condition (see also Figure A2). Bayes factors in favor of H0, *BF*_01_ > 3.00 (blue curly braces), occurred for the central bin for backward displacements and the two adjacent, ambiguous bins for forward displacements.

In sum, blanking during saccades improved jump estimates and this improvement did not come at the expense of false direction responses driven by noise: If the displacement was not missed (which happened less often), its direction was picked up correctly. In fact, blanking after saccades led to even better performance than blanking during fixation. This effect has already been reported by Deubel and colleagues [6], but has received little attention in the literature, but see [16]. The authors argued that the saccade brings the target close to the fovea where position signals are more accurate. The advantage seems to be greater than the error in the extraretinal signal. In fixation conditions, however, the comparison between two peripheral signals may introduce more spatial error. To my knowledge, the current data is the first to replicate the finding. Note, however, that the lack of replication for this saccade advantage up to date has a very simple reason: most (if not all) subsequent saccadic suppression of displacement studies examining the blanking effect did not include a fixation control condition [11,12,13,14,15,23,24,25,38].

Improvements with blanking in the masking conditions were smaller and restricted to forward displacements. Performance never exceeded the level from a 300 ms blank condition without mask (Appendix B), which is not surprising if one assumes that performance in the fixation condition reflects the precision and accuracy limits of peripheral vision. Also, on a small proportion of trials, the direction of small displacements was misjudged with masking, just as in fixation conditions without mask. Thus, it seems that just as Deubel and colleagues [6] proposed, comparing two peripheral signals is more error-prone than the comparison of a peripheral and a close-to-foveal signal, combined with extraretinal information about the saccade. 

## 4. General Discussion

When participants are asked to judge the ‘jump’ direction of a visual target, performance is poor when the displacement occurs simultaneously with a saccadic eye movement [2,3,4,5,6,7], an effect known as saccadic suppression of displacement. Prominent theories of saccadic suppression of displacement postulate that the poor performance is to a large part due to a default assumption or bias for stability across saccades [6,7,11,12,13,16]. However, not surprisingly, poor performance in displacement perception can also be observed without saccades, for instance, when the jump is obscured by a large pattern mask [30,31]. In the current experiments, I additionally examined continuous judgments of the displacement’s size and direction. Results suggest that saccade- as well as mask-induced suppression of displacement is primarily due to a larger proportion of trials on which participants report to have entirely missed the displacement (compared to a fixation control condition; Experiment 1). When introducing a 300 ms blank period straddling the jump, displacement detection was improved and the proportion of missed displacements reduced in the context of saccades and masks (Experiment 2). Complementing previous masking experiments [30,31], the current findings caution against the assumption of a saccade-specific stability bias.

### 4.1. A General Bias Revealed under Conditions of Impoverished Vision

The distributions of responses in the current experiments (and in particular the bimodality) support the idea of a stability bias or prior. Most simply put, small jumps are often missed across saccades and mask, but almost never in the control condition. In the saccadic context, this fits well with the idea that small discrepancies between prediction and incoming post-saccadic signal are discarded, in favor of a null hypothesis of stability [6,7,11,12,13]. It may also be in line with the more gradual influence of the bias and ‘contraction’ of jump estimates observed by Niemeier and colleagues [16,20]. It is important to note, however, that the masking conditions showed similar response distributions. This finding suggests that the bias for stability is not saccade-specific. 

Estimating the size of a displacement across saccades is challenged by two things: On the one hand, the visual system must integrate extraretinal signals or at least establish object correspondence across a large shift of the retinal image. This challenge is saccade-specific. On the other hand, visual perception is suppressed during saccades, as has been demonstrated in many previous studies, e.g., [27,28,29]. This challenge is not specific to situations of self-motion. Studies of saccadic suppression of displacement rarely made attempts to estimate the influence of those two challenges on performance, but see, e.g., [16,25,30,31]. Moreover, given that the context in which suppression of displacement is discussed is usually restricted to eye movements [6,7,11,12,13,14,15,16,19,20,22,23,24,25,26], one could have been tempted to conclude that the proposed mechanisms are saccade-specific. For instance, one may have assumed that the visual system adjusts the decision threshold for displacement perception across saccades to compensate for imprecisions introduced through the spatial updating mechanism. In other words, more evidence would be required across saccades for the perceptual null hypothesis to be rejected. Such a saccade-contingent adjustment provides indeed an intuitive account during eye movements. However, it is a less obvious explanation in the masking case when no spatial updating occurs. Alternatively, the current masking results suggest that it is not the decision threshold that is raised through saccades and masks, but that the visual evidence for a displacement is poorer. Thus, with smaller displacements, there is a higher probability that the amount of evidence remains below a general decision threshold, applied by the visual system across many situations and not specific to saccades. 

In the control condition of the current experiments, participants almost never missed displacements. It seems as if evidence for a displacement in the control condition is abundant, almost never below the threshold. (In line with this idea, small displacements were frequently overestimated: see Figure A1 in Appendix A.) How can saccades or masks so drastically impoverish this evidence? Note that the target itself is highly visible, see [30] for a visibility control experiment. Then again, not the target, but its displacement needs to be judged. It has been argued that saccades and masks both reduce the offset and onset transients usually perceived when the target jumps [6,25,30]. These transient signals alone might already facilitate the judgment of the displacement. Moreover, without strong transients, the impression of apparent motion that accompanies the jump should also be greatly reduced [6,25,30,33]. Perceived motion is a powerful cue, as it also conveys direction (which is all that is needed in a direction-discrimination task). Further, numerous illusions attest the influence of motion or apparent motion on position estimates [39,40,41]. In the control conditions, those signals are clearly seen and can reinforce the perception of a displacement. When degraded through saccades, masks or other circumstances, a crucial movement cue is lacking and thus displacements may be missed or underestimated. Indeed, Gysen and colleagues [22] have found that the detection of trans-saccadic jumps is better for a moving than a stationary object, demonstrating that violations along a smooth, predictable motion trajectory can be detected more easily than simple jumps. It is important to note that many authors have acknowledged that displacement estimates during fixation and saccades could rely on different sources of information: Whereas motion information is suppressed during saccades, apparent motion, as well as position information, is available during fixation [6,16,25,33,42]. For instance, saccadic suppression of displacement in the model by Niemeier and colleagues [16] is not due to a saccade-specific setting for the prior, but occurs due to a combination of suppressed motion signals (i.e., sole reliance on position information) and the Bayesian prior for jumps: The poorer the sensory information (e.g., due to motion suppression) the greater the influence of the Bayesian prior. Nevertheless, although a role for the quality of sensory information has been acknowledged, the idea of a stability bias has received much more attention in the saccadic suppression of displacement literature.

### 4.2. Improvement with Blanking: Relaxed Bias or Better Evidence?

Crucial for the prominent idea of a (variable) stability bias has been the improvement with blanking across saccades ([6] as replicated in Experiment 2). This counterintuitive finding has been taken as evidence that extraretinal signals seem to be more precise than initially assumed, but apparently not used in the non-blank conditions because of the default assumption of stability [6,7,11,12,13]. In blanking conditions, however, the stability bias is relaxed because the visual system has evidence that object continuity has been broken. Thus, it is argued, a concurrent change in position becomes, a priori, more likely as well, see also [16]. While the current findings do not speak against this account, an alternative could be that the blank does not change the bias, but actually improves the quality of the position signals. The blank makes it possible to perceive the onset transient upon reappearance of the displaced target in saccade as well as mask conditions. Again, this has not been overlooked in stability bias accounts. Quite to the contrary, the visible transient is thought to be one important source of evidence for a break in object continuity responsible for relaxing the bias [6,11,12,13,16]. Alternatively, the transient signal could actually improve displacement sensitivity. One possibility is that the transient enhances the precision of position signals, for instance, by engaging the magnocellular pathway [25]. In line with this assumption, it has been shown that reducing the onset’s strength by diminishing the target’s contrast to the background reduces or even abolishes the positive effect of blanking during saccades [25]. Another possibility is that the blank interval puts the post-saccadic or post-mask stimulus outside a critical temporal window for accumulation or integration of position or motion information [30,31,42,43]. Zimmermann and colleagues [31] argue that precise encoding of the pre-jump target location takes some time. The blank interval may provide that time. Further, they demonstrated that saccade- as well as mask-induced suppression of displacement is reduced when the pre-saccadic target is shown for a longer period of time before it is displaced. Others hypothesized that saccades, as well as masks, may lead to temporary distortions in the incoming position signal of the post-saccadic or post-mask target [44]. If the re-appearance of the target after the saccade or mask is delayed, though, the processes responsible for the distortions have already ceased and thus do not hamper displacement perception anymore. Additionally, blanking may also put pre- and post-jump target outside a critical temporal interval for perceiving apparent motion. This could explain why blanking during fixation (without mask) is detrimental to displacement perception ([6]; see also the additional Experiment 3 in Appendix B). Further, blanking has been found to be detrimental to transsaccadic displacement perception with moving objects [23], confirming that blanking interferes with the integration of motion information. 

Taken together, during fixation without any event or blank (control) we are most sensitive to displacements as the offset and onset transients of the target and apparent motion are clearly perceived (see Supplementary Video S1). Blanking during fixation probably reduces the motion signal somewhat (see Supplementary Video S4), but the strong offset and onset transients are preserved. Without blank, saccades and masks degrade the transients as well as the motion signal (see Supplementary Video S2). Blanking during saccades and masks makes at least the onset signal available and delays the post-jump target, thus improving performance in saccade and mask conditions (see Supplementary Video S3). To explain any of these modulations, assuming a relaxation in the stability bias is not necessary. Thus, the ‘bias’ may, in the end, reflect a general, fixed perceptual or decision threshold for displacements.

### 4.3. Saccade-Specific Effects?

One may argue that the current emphasis on the saccade context in the literature, although maybe slightly misleading, is not really problematic. After all, if saccadic suppression of displacement reveals a general stability bias that operates over a wide range of situations, it is, of course, legitimate to examine it exemplarily in the saccadic context. However, as mentioned before, perception across saccades does face the additional challenge of spatial updating. By not trying to disentangle effects due to spatial updating from the results of poor vision, we may actually miss the opportunity to measure truly saccade-specific effects.

In the current experiments, although masks had overall similar effects to saccades, only in the saccade condition did blanking improve performance up to a level that was actually better than in a blanking control condition (Experiment 2). Deubel and colleagues [6] had already observed that performance in saccadic blanking conditions can be better than in blanking conditions during fixation. They speculated that bringing the stimulus from the periphery into the fovea by means of the saccade improves the spatial representation of the target after displacement. Combined with information about the saccade amplitude, the judgment of the jump size could thus be more precise than when the target remains entirely in the periphery. This may also explain why blanking effects were weaker with masking, where stimuli remain in the periphery. Quite strikingly, there were only very few direction errors in the saccade conditions. This could suggest that some weak directional or motion signal survives across the saccade. Indeed, it has been shown that apparent motion, although weakened, can be perceived across saccades [45,46,47]. In the masking conditions, motion signals may be suppressed more thoroughly, which may also be the reason why masking produced more false direction responses. 

Further, it has now been repeatedly observed that not only blanking, but also a change in target features (e.g., polarity, shape or orientation) across the saccade results in weaker saccadic suppression of displacement [11,13,14,30]. The principal idea is again that the feature change lowers the decision threshold for displacement detection or attenuates a bias for stability. Because a change in the target is noticed across the saccade, concurrent differences in position signals are trusted. In principle, establishing object continuity or correspondence is a problem that arises in many situations, not only across saccades, e.g., [48,49]. The question is whether the visual system is more strongly biased to affirm spatial stability when object correspondence is established in the saccadic context. Here, I have argued against such saccade-specific settings. However, we have previously observed that an orientation change, although beneficial across saccades, did not improve performance across a mask [30] (see also Supplementary Experiment 4 for another potential dissociation). Future experiments may be needed to clarify whether the feature change effect is due to a saccade-specific bias setting, or, for instance, a difference in masking efficiency between saccades and our random-luminance mask.

Finally, even though saccadic suppression of displacement as such may not reveal much about how our own movements are integrated into visual experiences, this, of course, is not to say that those processes are not in effect in the paradigm. Quite to the contrary, if updating did not take place, we would not be able to estimate the displacement with such high precision across saccades (see Experiment 2; [6,17,18]). Interestingly, some studies have combined the suppression of displacement paradigm with manipulations that should affect the signals conveying motor information about the saccadic eye movement more directly. Of note, no matter whether the motor feedback (mostly, the corollary discharge was targeted) was altered through saccadic adaptation [17,50], transcranial magnetic stimulation over the frontal eye fields [51], a selective lesion of the thalamus [18], or inactivation of the assumed feedback route through muscimol [52], the resulting data pattern was always better characterized by a uniform shift in the psychometric function. It is thus maybe not so much a question of the experimental paradigm, but of the measure to be taken as an index for the processes involved in spatial stability and spatial updating across eye movements.

## 5. Materials and Methods

Data available on OSF: https://osf.io/z4pcm/.

### 5.1. Experiment 1

#### 5.1.1. Participants

Twenty-eight first-year psychology students (eight men) between 17 and 28 years of age completed Experiment 1 in a single one-hour session. They reported normal or corrected-to-normal vision and received course credit for their participation. For all experiments in this study, observers gave written informed consent prior to participating and the procedures followed the principles laid down in the Code of Ethics of the World Medical Association (Declaration of Helsinki) and were approved by the Ethics committee of the *Faculté de Psychologie et des Sciences de l’Education* of the University of Geneva (Project submitted: PZ00P1_161224, entitled “Space without motion—judging object locations and relative distance in the absence of movement”; date of acceptance: February 19, 2016). 

#### 5.1.2. Apparatus

Experiments were programmed in Matlab (The MathWorks Inc., Natick, MA, USA) using the Psychophysics and Eyelink Toolbox extensions [53,54]. Stimuli were displayed on a 21″ CRT monitor (NEC MultiSync FE2111SB) running at 85 Hz with a resolution of 1280 × 1024 pixels. Eye movements were recorded using an EyeLink1000 desk-mounted eye tracker (SR Research Ltd., Ottawa, ON, Canada) at a sampling rate of 1000 Hz. Participants were seated in a dimly lit room. Viewing was binocular but only the right eye was monitored. The participant’s head was stabilized by a chin and a forehead rest at 45 cm from the monitor.

#### 5.1.3. Stimuli, Design and Procedure

The procedure in Experiment 1 is illustrated in Figure 1A. Stimuli were displayed on a gray background (16 cd/m^2^). Each trial started with the presentation of a black fixation square (subtending 0.5 deg) at the screen center. After 1000 ms, the red vertical target bar (9 cd/m^2^, 0.6 × 3 deg) appeared either left or right from fixation on the horizontal meridian. Target eccentricity from fixation was on average 10 deg, but a random jitter was added on each trial, such that eccentricity ranged between 8 and 12 deg. In the fixation blocks, the target remained at its initial position for 200 ms and then was either followed by a 50 ms blank screen (control condition) or a 50 ms mask (full-screen pattern mask, made up of gray squares of 0.5 deg side length and randomized luminance). Then, the target bar was displaced (it ‘jumped’) randomly either leftward or rightward (possible displacement sizes: 0.5, 1, 2 or 3 deg). Participants were required to maintain gaze on the central fixation square until the displacement had occurred. Then, they were free to move their eyes. In the saccade blocks, participants were instructed to make an eye movement towards the red target bar upon its appearance. It was displaced as soon as a saccade was detected. In both fixation and saccade blocks, the target bar in its final position was shown as a reference until participants gave a response: They were asked to report the direction and size of the target jump by indicating the displacement’s starting point with respect to its endpoint through a mouse click on the respective location on the screen. A feedback message was displayed on the response screen if a fixation or saccade error was registered during the trial (see Section 5.3 below for criteria). In total, participants completed three blocks of 160 trials: They always started with a fixation block (including randomly interleaved control and masking trials), then ran one saccade block and finished with a second fixation block. Trials with the target presented to the left or right from fixation were collapsed and left/right displacements recoded into ‘forward’ (jump further out into the periphery), and ‘backward’ (jump back towards the initial fixation location at the center of the screen). For each combination of condition (control, saccade, mask), displacement size and direction, 20 data points were collected from each participant.

### 5.2. Experiment 2

Another twenty-two first-year psychology students (six men) between 18 and 45 years of age completed Experiment 2 in a single one-hour session. Apparatus, stimuli, design and procedure were similar to Experiment 1, but there was no control condition without saccades or masks. Instead, saccade and masking trials without blank were compared to conditions in which a 300 ms blank followed the mask or saccade (see Figure 1B). Participants completed four blocks of 144 trials: Two blocks in the saccade, and two blocks in the masking condition in random order. Within these blocks, blank and no blank trials were randomly interleaved across trials, resulting in 18 data points per combination of condition (mask vs. saccade), blanking (no blank vs. 300 ms blank) and displacement size and direction.

### 5.3. Saccade Detection and Trial Exclusion Criteria for Both Experiments

In saccade conditions, the displacement was initialized as soon as the horizontal gaze coordinate taken online deviated by more than 1.5 deg from an initial sample taken at target onset of each trial. After the experiment, fixation periods, saccade onsets, offsets and blinks were extracted offline from the data of all conditions using the Eyelink parser with a velocity criterion of 30°/s and an acceleration criterion of 8000°/s^2^ for saccades. Trials in fixation conditions (control or mask) were discarded if a saccade was detected or the horizontal gaze coordinate drifted more than 1.5 deg away from screen center in a time window from 100 ms before target onset until 100 ms after the jump (breaks of fixation: 10.9% of trials in Experiment 1, 11.1% of trials in Experiment 2), or when a blink was detected during the same time window (3.1% of trials in Experiment 1, 2.0% in Experiment 2). Taken together, 12.0% of trials were discarded as errors from the fixation blocks (control or mask) in Experiment 1, and 12.1% of trials were discarded from the fixation (mask) blocks in Experiment 2. In saccade conditions, trials were excluded if gaze at the time of saccade onset deviated more than 1.5 deg from the screen center (breaks of fixation: 5.7% in Experiment 1, 5.4% in Experiment 2), if a saccade was detected less than 100 ms after target onset (anticipations: 3.7% in Experiment 1, 5.6% in Experiment 2), if the saccade was made into the wrong direction (0.8% in Experiment 1, 1.0% in Experiment 2), if the saccade amplitude was smaller than 5 deg (2.2% in Experiment 1, 3.2% in Experiment 2), if no saccade was detected within 500 ms of target onset (11.5% in Experiment 1, 7.8% in Experiment 2), or when a blink occurred (3.3% in Experiment 1, 2.2% in Experiment 2). Taken together, 20.8% of trials were discarded from the saccade blocks in Experiment 1, and 19.6% of trials from the saccade blocks in Experiment 2. Saccades were executed with an average latency of 236 ms in Experiment 1, and 219 ms in Experiment 2. The displacement occurred on average 28 ms after saccade onset and 22 ms before saccade offset in Experiment 1. In Experiment 2, the displacement occurred on average 29 ms after saccade onset and 22 ms before saccade offset. Average saccade amplitude was 9.21 deg in Experiment 1 and 9.62 deg in Experiment 2.

## Figures and Tables

**Figure A3:**
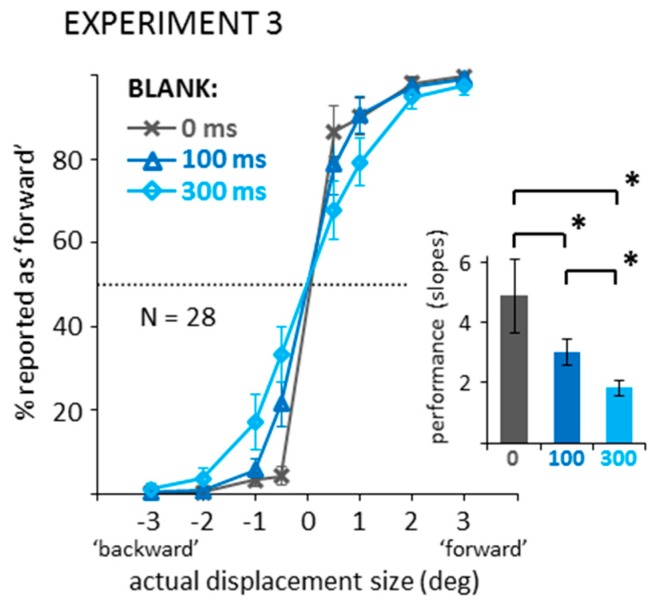
Data recoded into binary displacement direction responses. A: Data from the 0, 100, and 300 ms blank during fixation conditions of Experiment 3. Line graph shows actual displacements (negative: ‘backward’ vs. positive: ‘forward’) against the percentage of reported ‘forward’ responses (dotted line: chance level). Bar graph shows average slopes of the logistic functions fitted to each participant’s individual data. All error bars: between-subjects 95% confidence intervals. Brackets marked with * illustrate significant differences in post-hoc paired-samples *t*-tests (*p* < 0.05).

**Figure A4:**
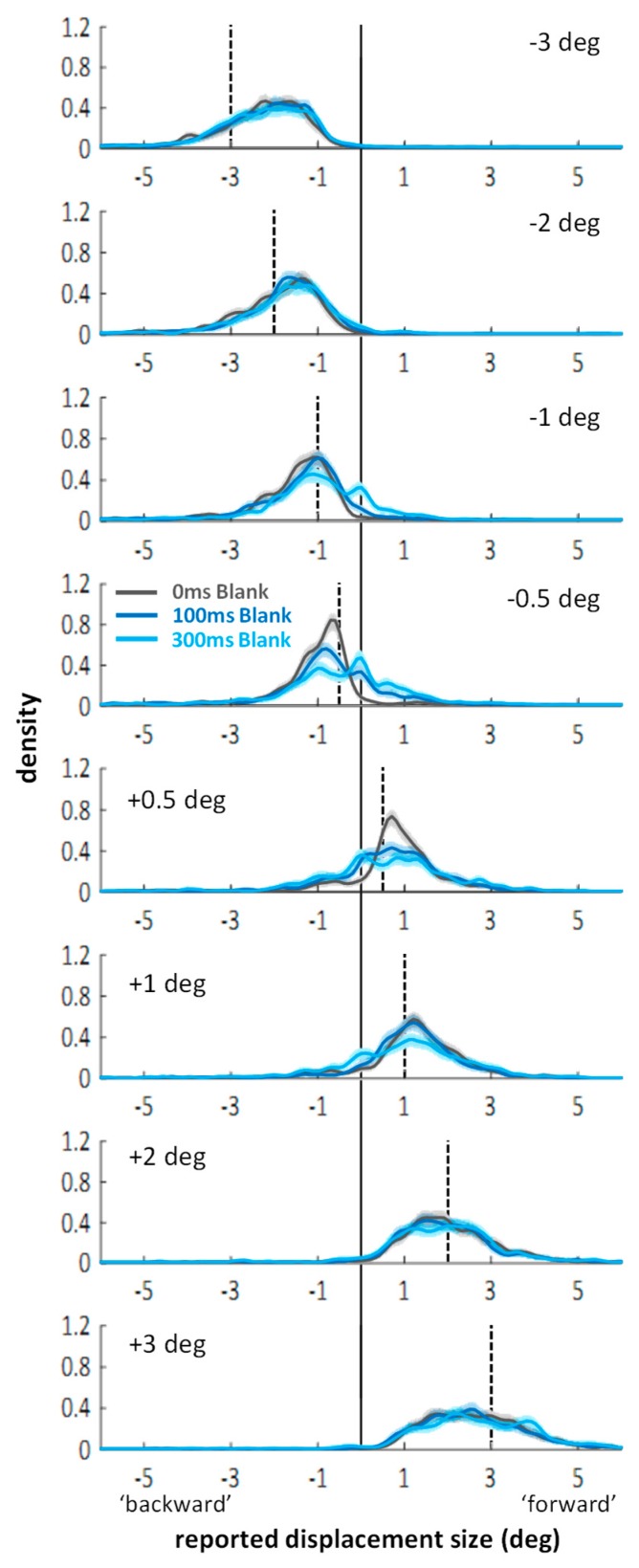
Distribution of responses (kernel density functions) in the three conditions of Experiment 3 (color-coded). Rows represent the different displacement sizes tested. Data are collapsed across all 28 participants. Shadings represent bootstrapped 95% confidence intervals. Vertical black continuous line: zero line. Vertical black dashed lines: actual displacement for each graph. Overall, distributions in the 0 ms blank control condition resembled the ones from Experiment 1 (except that overestimations of smaller displacements seemed somewhat smaller; as for Experiment 1, almost no misses occurred, though). In the 100 and 300 ms blank conditions, some of the smaller ±0.5 and ±1 deg displacements were missed. In the 300 ms blank conditions, this is visible in small peaks around zero, while the 100 ms blank conditions rather showed a spread of the distribution towards zero, compared to the no blank condition.

## Supplementary Materials

The following are available online at https://www.mdpi.com/2411-5150/3/4/49/s1, Video S1: Control, Video S2: Mask, Video S3: Blank, Mask, Video S4: Blank, Control, Supplementary File S5: Experiment 4.

## Appendix B. Experiment 3—Displacement Estimates in Blanking Conditions without Saccades or Mask (Control for Experiment 2)

Blanking effects have mostly been studied in saccade conditions as they produce a somewhat surprising increase in discrimination performance. Less studied has been the finding that in fixation conditions, performance decreases when a blank is introduced between the offset of the target at its initial position and its onset at the final position [6]. Demo movies of the 0 ms blank (Video S1) and the 300 ms blank (Video S4) conditions can be found in Supplementary Material online.

### Appendix B.1. Methods

Another twenty-eight psychology students (sixteen women) between 18 and 45 years of age completed Experiment 3 in a single one-hour session. Apparatus, stimuli, design and procedure were similar to the control condition of Experiment 1. Instead of comparing control, mask and saccade conditions, I compared three different blanking intervals (0, 100, and 300 ms) between the target’s offset at its initial position and its onset at the final position. Participants completed three blocks of 168 trials. The blanking conditions were randomly interleaved across trials in all three blocks. As in the fixation blocks of Experiment 1, participants were required to maintain gaze on the central fixation square during stimulus presentation and this was monitored by means of the eyetracker. For each combination of blanking condition (0, 100, 300 ms), displacement size and direction, 21 data points were collected from each participant.

### Appendix B.2. Results and Discussion

#### Appendix B.2.1. Direction Discrimination: Suppression of Displacement (Recoded Data)

Again, data was first recoded into binary responses and the percentage of reported ‘forward’ jumps for each displacement direction and size was calculated. Figure A3 illustrates the results and shows average slopes of the logistic functions fitted to the data of each individual observer. Slopes were less steep with longer blanking intervals. A one-way ANOVA confirmed a significant main effect, *F*(2,54) = 20.03, *p* < 0.001, partial *η*^2^ = 0.426 and subsequent paired-samples *t*-tests revealed significant differences between all conditions, *t*s(27) > 3.19, *p*s < 0.004, *BF*_10_ > 11.08. Thus, we see significantly worse performance with increasing blank interval in the fixation control condition. Numerically, the performance decrease was, however, much smaller compared to the saccade and mask conditions in Experiment 1, which is in line with previous results [6]. Figure A4 additionally shows the distributions of responses separate for each displacement size.

## References

[1] Simons D.J., Rensink R.A. (2005). Change blindness: Past, present, and future. Trends Cogn. Sci..

[2] Wallach H., Lewis C. (1966). The effect of abnormal displacement of the retinal image during eye movements. Percept. Psychophys..

[3] Mack A. (1970). An investigation of the relationship between eye and retinal image movement in the perception of movement. Percept. Psychophys..

[4] Bridgeman B., Hendry D., Stark L. (1975). Failure to detect displacement of the visual world during saccadic eye movements. Vis. Res..

[5] Li W.X., Matin L. (1990). The influence of saccade length on the saccadic suppression of displacement detection. Percept. Psychophys..

[6] Deubel H., Schneider W.X., Bridgeman B. (1996). Postsaccadic target blanking prevents saccadic suppression of image displacement. Vis. Res..

[7] McConkie G.W., Currie C.B. (1996). Visual stability across saccades while viewing complex pictures. J. Exp. Psychol. Hum. Percept. Perform..

[8] Bridgeman B., Van der Heijden A.H.C., Velichkovsky B.M. (1994). A theory of visual stability across saccadic eye movements. Behav. Brain Sci..

[9] Grüsser O.J. (1994). Early concepts on efference copy and reafference. Behav. Brain Sci..

[10] Li W.X., Matin L. (1990). Saccadic suppression of displacement: Influence of postsaccadic exposure duration and of saccadic stimulus elimination. Vis. Res..

[11] Tas A.C., Moore C.M., Hollingworth A. (2012). An object-mediated updating account of insensitivity to transsaccadic change. J. Vis..

[12] Wexler M., Collins T. (2014). Orthogonal steps relieve saccadic suppression. J. Vis..

[13] Demeyer M., De Graef P., Wagemans J., Verfaillie K. (2010). Object form discontinuity facilitates displacement discrimination across saccades. J. Vis..

[14] Poth C.H., Herwig A., Schneider W.X. (2015). Breaking object correspondence across saccadic eye movements deteriorates object recognition. Front. Syst. Neurosci..

[15] Irwin D.E., Robinson M.M. (2018). How post-saccadic target blanking affects the detection of stimulus displacements across saccades. Vis. Res..

[16] Niemeier M., Crawford J.D., Tweed D.B. (2003). Optimal transsaccadic integration explains distorted spatial perception. Nature.

[17] Collins T., Rolfs M., Deubel H., Cavanagh P. (2009). Post-saccadic location judgments reveal remapping of saccade targets to non-foveal locations. J. Vis..

[18] Ostendorf F., Liebermann D., Ploner C.J. (2010). Human thalamus contributes to perceptual stability across eye movements. Proc. Natl. Acad. Sci. USA.

[19] Currie C.B., McConkie G.W., Carlson-Radvansky L.A., Irwin D.E. (2000). The role of the saccade target object in the perception of a visually stable world. Percept. Psychophys..

[20] Niemeier M., Crawford J.D., Tweed D.B. (2007). Optimal inference explains dimension-specific contractions of spatial perception. Exp. Brain Res..

[21] MacKay D.M., Jung R. (1973). Visual stability and voluntary eye movements. Central Processing of Visual Information A: Integrative Functions and Comparative Data.

[22] Gysen V., De Graef P., Verfaillie K. (2002). Detection of intrasaccadic displacements and depth rotations of moving objects. Vis. Res..

[23] Gysen V., Verfaillie K., De Graef P. (2002). The effect of stimulus blanking on the detection of intrasaccadic displacements of translating objects. Vis. Res..

[24] Irwin D.E., Robinson M.M. (2015). Detection of stimulus displacements across saccades is capacity-limited and biased in favor of the saccade target. Front. Syst. Neurosci..

[25] Matsumiya K., Sato M., Shioiri S. (2016). Contrast dependence of saccadic blanking and landmark effects. Vis. Res..

[26] Atsma J., Maij F., Koppen M., Irwin D.E., Medendorp W.P. (2016). Causal inference for spatial constancy across saccades. PLoS Comput. Biol..

[27] Breitmeyer B.G., Ganz L. (1976). Implications of sustained and transient channels for theories of visual pattern masking, saccadic suppression, and information processing. Psychol. Rev..

[28] Burr D.C., Morrone M.C., Ross J. (1994). Selective suppression of the magnocellular visual pathway during saccadic eye movements. Nature.

[29] Matin E. (1974). Saccadic suppression: A review and an analysis. Psychol. Bull..

[30] Zimmermann E., Born S., Fink G.R., Cavanagh P. (2014). Masking produces compression of space and time in the absence of eye movements. J. Neurophysiol..

[31] Zimmermann E., Morrone M.C., Burr D.C. (2013). Spatial position information accumulates steadily over time. J. Neurosci..

[32] Breitmeyer B.G., Öğmen H. (2006). Visual Masking: Time Slices through Conscious and Unconscious Vision.

[33] Shioiri S., Cavanagh P. (1989). Saccadic suppression of low-level motion. Vis. Res..

[34] Bays P.M., Catalao R.F., Husain M. (2009). The precision of visual working memory is set by allocation of a shared resource. J. Vis..

[35] Zhang W., Luck S.J. (2008). Discrete fixed-resolution representations in visual working memory. Nature.

[36] Rouder J.N., Speckman P.L., Sun D., Morey R.D., Iverson G. (2009). Bayesian *t* tests for accepting and rejecting the null hypothesis. Psychon. Bull. Rev..

[37] JASP (Version 0.8.2). Computer Software. https://jasp-stats.org/.

[38] Weiss K., Schneider W.X., Herwig A. (2015). A “blanking effect” for surface features: Transsaccadic spatial-frequency discrimination is improved by postsaccadic blanking. Atten. Percept. Psychophys..

[39] Whitney D. (2002). The influence of visual motion on perceived position. Trends Cogn. Sci..

[40] Li H.H., Shim W.M., Cavanagh P. (2014). Backward position shift in apparent motion. J. Vis..

[41] Kerzel D., Gegenfurtner K.R. (2004). Spatial distortions and processing latencies in the onset repulsion and frohlich effects. Vis. Res..

[42] Bergelt J., Hamker F.H. (2016). Suppression of displacement detection in the presence and absence of eye movements: A neuro-computational perspective. Biol. Cybern..

[43] Cicchini G.M., Binda P., Burr D.C., Morrone M.C. (2013). Transient spatiotopic integration across saccadic eye movements mediates visual stability. J. Neurophysiol..

[44] Ziesche A., Bergelt J., Deubel H., Hamker F.H. (2017). Pre- and post-saccadic stimulus timing in saccadic suppression of displacement—A computational model. Vis. Res..

[45] Szinte M., Cavanagh P. (2011). Spatiotopic apparent motion reveals local variations in space constancy. J. Vis..

[46] Fracasso A., Caramazza A., Melcher D. (2010). Continuous perception of motion and shape across saccadic eye movements. J. Vis..

[47] Rock I., Ebenholtz S. (1962). Stroboscopic movement based on change of phenomenal rather than retinal location. Am. J. Psychol..

[48] Anstis S. (1980). The perception of apparent movement. Philos. Trans. R. Soc. Lond. B Biol. Sci..

[49] Enns J.T., Lleras A., Moore C.M., Nijhawan R., Khurana B. (2010). Object updating: A force for perceptual continuity and scene stability in human vision. Space and Time in Perception and Action.

[50] Klingenhoefer S., Bremmer F. (2011). Saccadic suppression of displacement in face of saccade adaptation. Vis. Res..

[51] Ostendorf F., Kilias J., Ploner C.J. (2012). Theta-burst stimulation over human frontal cortex distorts perceptual stability across eye movements. Cereb. Cortex.

[52] Cavanaugh J., Berman R.A., Joiner W.M., Wurtz R.H. (2016). Saccadic corollary discharge underlies stable visual perception. J. Neurosci..

[53] Kleiner M., Brainard D., Pelli D. (2007). What’s new in psychtoolbox-3?. Perception.

[54] Cornelissen F.W., Peters E.M., Palmer J. (2002). The eyelink toolbox: Eye tracking with matlab and the psychophysics toolbox. Behav. Res. Methods Instrum. Comput..

