# Supplementary material for: Saccadic Suppression of Displacement Does Not Reflect a Saccade-Specific Bias to Assume Stability
Source: Vision (Basel). 2019 Sep 24;3(4):49. doi: 10.3390/vision3040049 (PMC6969937; doi:10.3390/vision3040049)
Supplement: Supplementary file 1 [file vision-03-00049-s001.zip › BornSupplement/BornSupplement_Experiment4.pdf]

vision

MDPI

Supplementary material to:

Saccadic suppression of displacement does not reflect a saccade-specific bias to assume stability

Sabine Born

1. Experiment 4

Target blanking is not the only condition that has been found to improve displacement perception across saccades. Changing the target's shape, polarity, object identity or orientation likewise produces better direction discrimination [1-4]. In Zimmermann and colleagues [4], we have found that an orientation change does not improve performance in a masking condition, though. Further, Wexler & Collins [5] report that across saccades, the horizontal component of oblique displacements can be better discriminated than an equivalent 'pure' horizontal displacement (i.e., without an additional vertical component). In Experiment 4, I adopted the approach of Wexler & Collins [5] and tested perception of horizontal and oblique displacements in control vs. mask conditions in one group, and control vs. saccade conditions in a second group. Before analysis, 11.0% of trials were discarded in the control conditions (both groups combined), 4.8% of trials in the mask condition, and 13.5% of trials in the saccade condition (see main manuscript for for criteria).

1.1 Methods

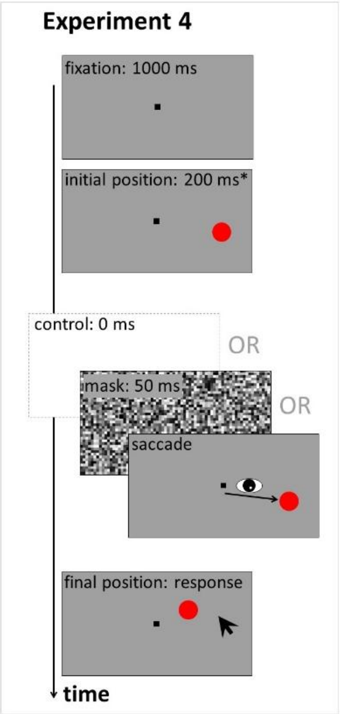

Supplementary Figure S1. Procedure in Experiment 4.

Thirty first-year psychology students completed Experiment 4. They were split into two groups (saccade vs. mask). Each participant completed four blocks of 144 trials: two blocks in a control condition (no saccade, no mask) and two blocks in either the saccade or the mask condition. Block order was randomized for each participant. The saccade group consisted of 15 participants (eight women) between 19 and 35 years of age, the mask group consisted of 15 participants (eight women) aged between 18 and 24 years. The sequence of events is illustrated in Supplementary Figure S1. Apparatus, stimuli, design and procedure were similar to the previous experiments, with the following exceptions: the red target bar was replaced by a red dot (1.2 deg in diameter). On half the trials in each block, this dot jumped only horizontally just like the bar target in the other experiments. On the other half of trials, the dot jumped along an oblique trajectory. The horizontal component of these oblique displacements was chosen from one of the jump sizes previously used (-3, -2, -1, -0.5, +0.5 +1, +2, or +3 deg). The vertical component was fixed to 1.2 deg, randomly either upward or downward. Targets appeared initially at an eccentricity of around 10 deg either left or right from fixation close to the horizontal meridian. However, to vary its starting position across trials, a random jitter between -2 deg and +2 deg was not only added horizontally (just like in the previous experiments), but also vertically, but only ranging between -1 deg (below the horizontal meridian) and +1deg (above the meridian). This vertical jitter in the starting position

was added on all trials (i.e., trials with pure horizontal displacements as well as trials with oblique displacements). Horizontal and oblique displacements were randomly interleaved in a block. For each combination of condition (control vs. saccade or mask), displacement type (horizontal vs. oblique) and jump size and direction, 18 data points were collected from each participant.

1.2 Results

1.2.1. Direction discrimination: Suppression of displacement (recoded data)

Supplementary Figure S2 illustrates the results from the two groups when recoding the data into binary responses. In the control condition of both groups, oblique jumps produced slightly shallower slopes, that is, worse performance when judging the horizontal component of the oblique displacement compared to the purely horizontal jumps. In the mask condition, oblique jumps also produced a slightly shallower curve. In the saccade condition, however, the curve was slightly steeper for oblique displacements. First, an ANOVA on the slopes of the individual psychometric functions (see bar graphs in Figure 9) was conducted, including the factor group (mask vs. saccade) as between-subject factor, and condition (control vs. mask/saccade) and displacement type (horizontal vs. oblique) as within-subject factors. All main effects and interactions were significant or close to significant, all *F*s > 3.47, *p*s < .073, partial *η2s* > .110. Most importantly, the three-way interaction between group, condition and displacement type was significant, $F(1,28) = 7.73$, $p = .010$, partial $\eta2 = .216$. Following up on this three-way interaction, I next conducted separate ANOVAs for the two groups. For the masking group, this post-hoc analysis revealed significant main effects of condition, $F(1,14) = 103.96$, $p < .001$, partial $\eta2 = .881$, and displacement type, $F(1,14) = 4.63$, $p = .049$, partial $\eta2 = .248$, confirming better performance in the control compared to the mask condition and worse performance for oblique compared to horizontal jumps. The two-way interaction was not significant, $F(1,14) = 0.25$, $p = .625$, partial $\eta2 = .018$. In contrast, in the saccade condition, not only the main effects of condition, $F(1,14) = 49.58$, $p < .001$, partial $\eta2 = .780$, and displacement type, $F(1,14) = 8.17$, $p = .013$, partial $\eta2 = .369$, were significant, but also the interaction, $F(1,14) = 10.20$, $p = .007$, partial $\eta2 = .421$. Subsequent Wilcoxon signed-rank tests confirmed worse performance with oblique displacements in the control condition, $p = .005$, and better performance with oblique displacements in the saccade condition, $p = .015$, replicating Wexler & Collins' [5] findings.

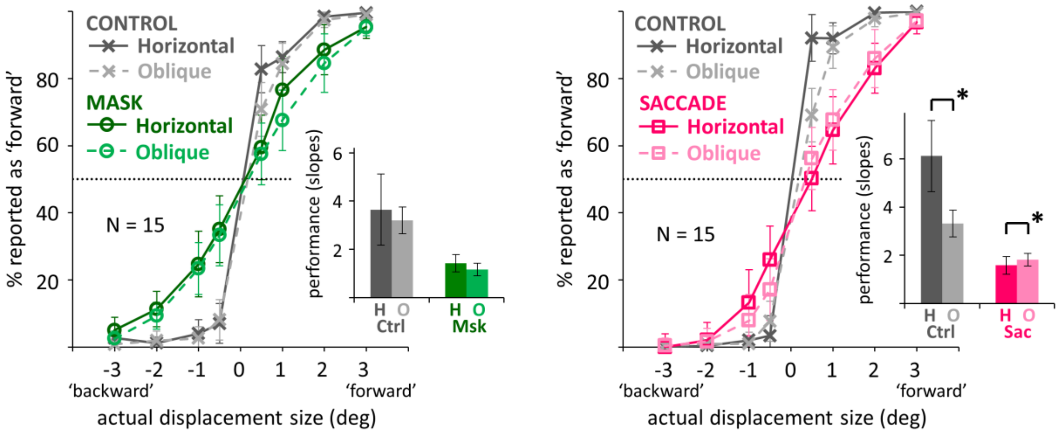

Supplementary Figure S2. Data from Experiment 4, recoded into binary displacement direction responses. Left: Data from the mask group across condition (control vs. mask), displacement type (horizontal vs. oblique) and displacement size. Right: Data from the saccade group across condition (control vs. saccade), displacement type (horizontal vs. oblique) and displacement size. Actual displacements and participant's responses in the oblique conditions refer solely to the horizontal component of the oblique displacement.

1.2.2. Distributions of displacement estimates

Supplementary Figures S3 and S4 show the distribution of responses as kernel density estimates. The left column of both figures show the control condition and compare judgments for pure horizontal displacements and the horizontal component of the oblique displacements. For oblique displacements, distributions were overall slightly shifted towards zero for almost all displacements. Further, in the control condition of the saccade group (Figure S4), the distributions for the +0.5 deg and +1 deg oblique displacements also show some hints of bimodality. It is important to recall that the judgments here only consider the horizontal component of an oblique movement with an additional ±1.2 deg vertical component. This vertical component makes it unlikely that the displacement is completely missed in the control condition. A click around zero would thus not indicate a miss, but a purely vertical movement. One can easily imagine that the visual system relies on some kind of threshold mechanism to determine whether a stimulus has moved or not. It is unlikely that the same threshold is applied for isolating the horizontal component out of an oblique movement.

The right column of Figures S3 and S4 illustrate the distribution of responses in the mask and saccade conditions. In the judgments of horizontal displacements, just like in the previous experiments, we see bimodal distributions with peaks around zero indicating missed displacement, at least for the smaller displacements. Those seem again more pronounced for saccades than masks.

In the mask conditions (Figure S3, right column), oblique displacements produce only very subtle differences in the distributions compared to horizontal displacements. If anything, it seems the peaks and dips are smoothed out somewhat. Both masking conditions also produced small proportions of false direction responses as can be seen in long tails spreading towards the wrong side, especially for the smaller displacements (best seen at -0.5 deg). The thin gray lines illustrate the distributions from the oblique control condition for comparison. Combining oblique displacements with masking had a detrimental effect: as can be seen from the spread and the location of the distributions, responses in the control condition are more precise and more accurate.

When looking at the saccade conditions in Figure S4 (right column), a similar picture emerges. The oblique displacement condition produces only small differences in the distributions compared to the purely horizontal displacements. One may again say that peaks and troughs are smoothed somewhat. The slightly diminished peaks around zero perceived displacements may be seen as small improvements. Especially for -0.5 deg displacements, the peak around zero is markedly reduced and the backward direction of the jumps is clearly picked up more often in the oblique condition than in the horizontal condition. This difference (along with the more subtle differences in other conditions) seems to be the main driving force for the significant improvement in the slope of the psychometric function in the saccade condition (see Supplementary Figure S3). Note, however that in contrast to the blanking effect, performance in the saccade conditions was never better than in the control condition. To the contrary, more precise and/or more accurate estimates were given in the control condition.

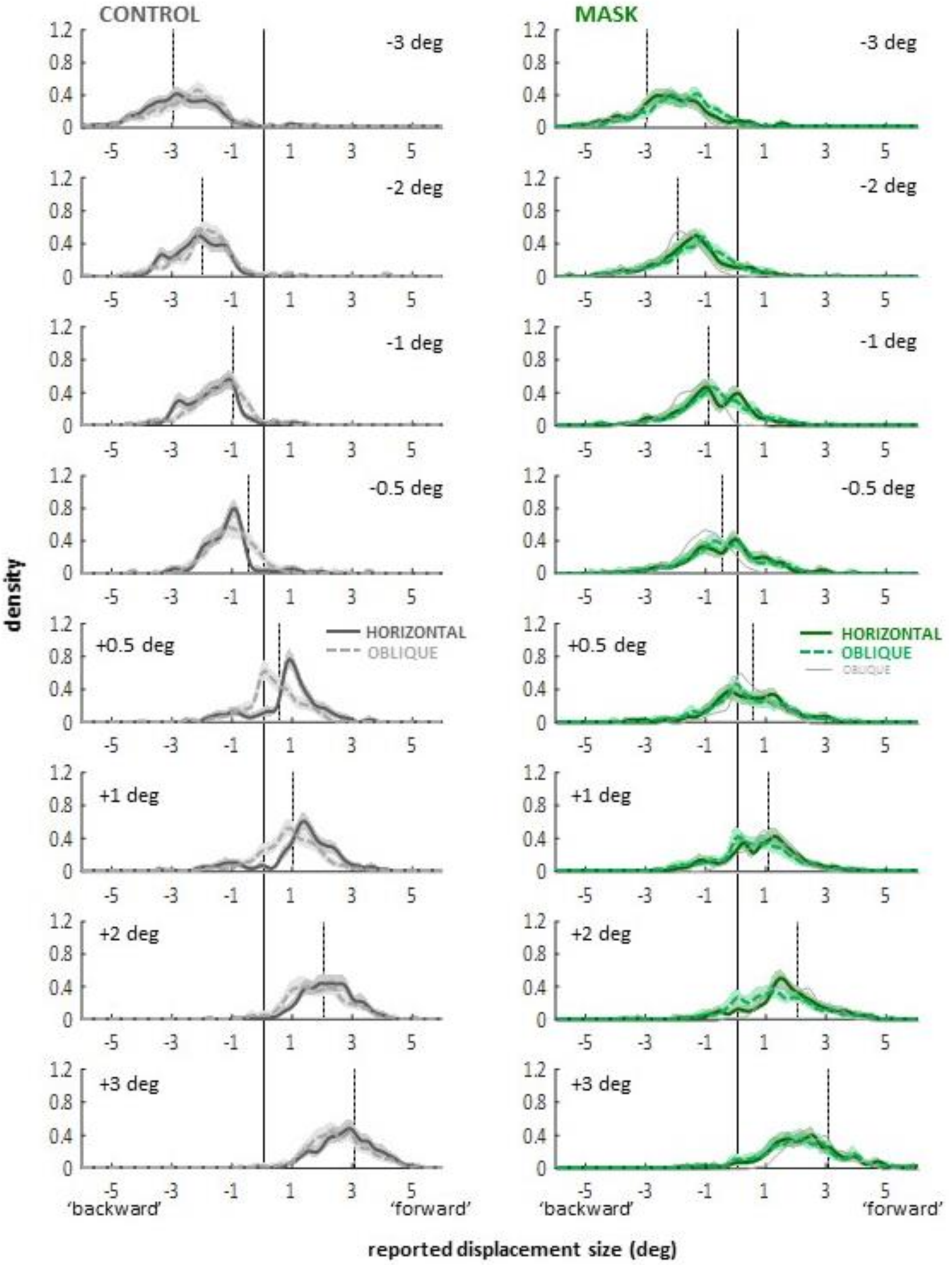

Supplementary Figure S3. Distribution of responses (kernel density functions) for the mask group of Experiment 4. Responses for the horizontal (continuous lines) and oblique (dashed lines) displacements in control (left) and mask (right) conditions. For oblique displacements only the horizontal component of displacement and response is considered here.

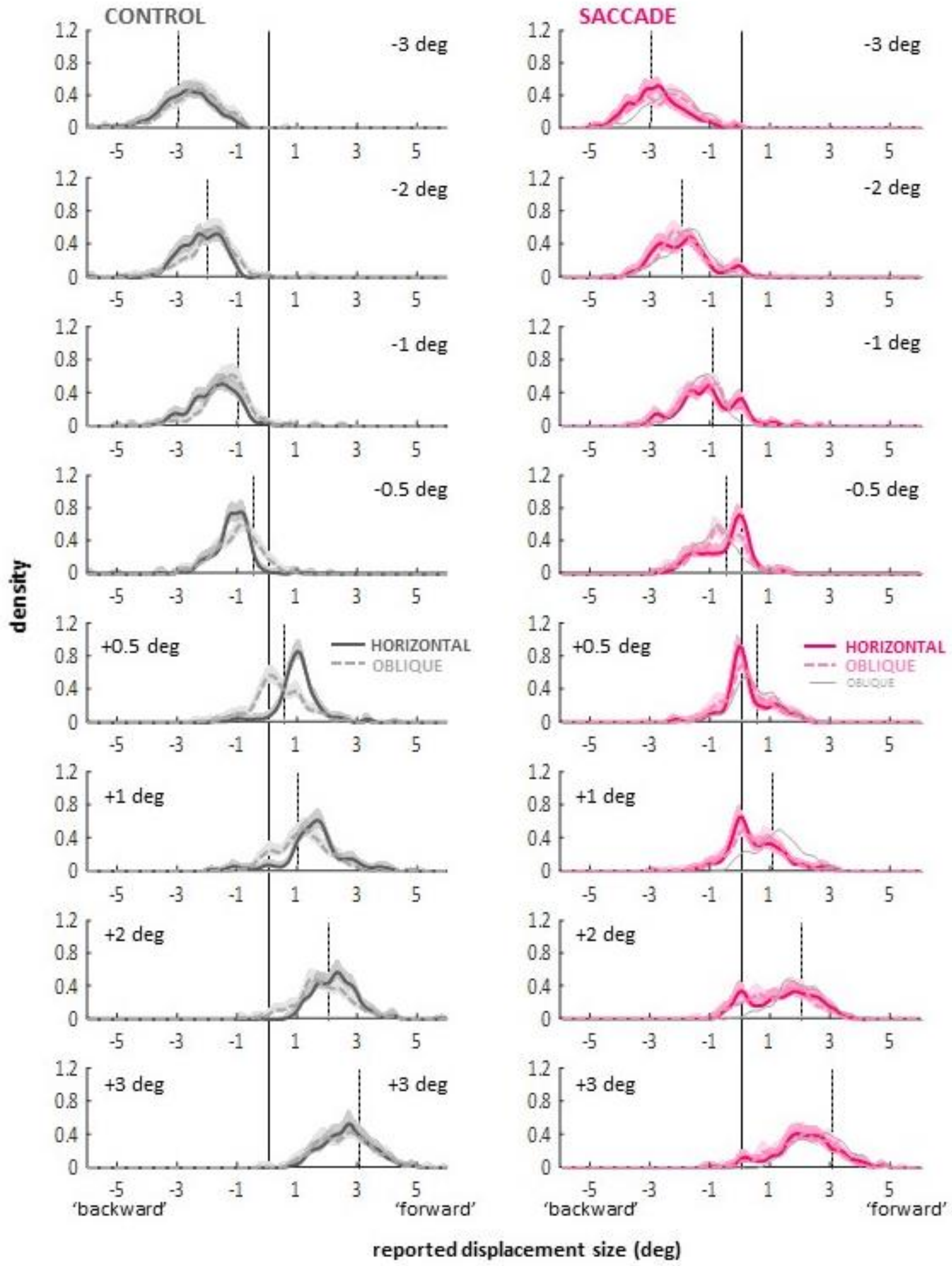

Supplementary Figure S4. Distribution of responses (kernel density functions) for the saccade group of Experiment 4. Responses for the horizontal (continuous lines) and oblique (dashed lines) displacements in control (left) and saccade (right) conditions.

1.3 Discussion

In sum, the improvement in direction discrimination with oblique displacements described by Wexler and Collins [5] in the saccade condition was replicated. However, there was no improvement in the masking condition and worse performance for oblique compared to pure horizontal displacements in the control condition. The effect for saccades was in general small, except for -0.5 deg backward displacements. In contrast, Wexler and Collins [5] report improvements quite similar in strength to a blanking procedure. One difference between studies is that the stimuli in the current experiment were presented slightly further out in the periphery: The initial position here was between 8 and 12 deg eccentricity, whereas Wexler and Collins [5] presented their targets initially at 6 or 8 deg. Thus, larger improvements (also visible for more displacement levels) could be possible at smaller eccentricities. The relatively small number of participants per group may also have played a role. Although 15 participants per group was similar to Wexler and Collins (who present data from 13 participants [5]), the mouse pointing technique used here allows for more variability in responses. Thus, even recoded data may produce smaller modulations across conditions, and the amount of data may have been insufficient to see stronger modulations in the distributions. Thus, as results rely on comparatively small modulations, Experiment 4 is only presented as supplementary material.

Taken at face value, however, the improvement for judging the horizontal component of oblique displacements compared to pure horizontal displacements across saccades may present another example of a saccadic effect that cannot be mimicked with a stationary full-screen random luminance mask. It remains an open question, though, whether this reflects a saccadic-specific stability bias, or a weaker masking effect of the saccade. As has been mentioned in the main manuscript, apparent motion, although weakened, can be perceived across saccades [6-8]. The additional vertical component of oblique displacements may change the perceived motion in a way that made it possible to sometimes detect the slight horizontal offset (e.g., by introducing the perception of a small tilt in the apparent motion trajectory) in the saccade condition. In the masking conditions, motion signals may be suppressed more thoroughly, precluding any benefit from oblique displacements.

References

1. Tas, A.C.; Moore, C.M.; Hollingworth, A. An object-mediated updating account of insensitivity to transsaccadic change. *J Vis* **2012**, *12*, 18, DOI: 10.1167/12.11.18.
2. Demeyer, M.; De Graef, P.; Wagemans, J.; Verfaillie, K. Object form discontinuity facilitates displacement discrimination across saccades. *J Vis* **2010**, *10*, 17, DOI: 10.1167/10.6.17.
3. Poth, C.H.; Herwig, A.; Schneider, W.X. Breaking object correspondence across saccadic eye movements deteriorates object recognition. *Frontiers in systems neuroscience* **2015**, *9*, 176, DOI: 10.3389/fnsys.2015.00176.
4. Zimmermann, E.; Born, S.; Fink, G.R.; Cavanagh, P. Masking produces compression of space and time in the absence of eye movements. *J. Neurophysiol.* **2014**, *112*, 3066-3076, DOI: 10.1152/jn.00156.2014.
5. Wexler, M.; Collins, T. Orthogonal steps relieve saccadic suppression. *J Vis* **2014**, *14*, DOI: 10.1167/14.2.13.
6. Szinte, M.; Cavanagh, P. Spatiotopic apparent motion reveals local variations in space constancy. *J Vis* **2011**, *11*, DOI: 10.1167/11.2.4.
7. Fracasso, A.; Caramazza, A.; Melcher, D. Continuous perception of motion and shape across saccadic eye movements. *J Vis* **2010**, *10*, 14, DOI: 10.1167/10.13.14.
8. Rock, I.; Ebenholtz, S. Stroboscopic movement based on change of phenomenal rather than retinal location. *Am. J. Psychol.* **1962**, *75*, 193-207, DOI: 10.2307/1419603.
