# Supplementary figures and images for: Saccadic Suppression of Displacement Does Not Reflect a Saccade-Specific Bias to Assume Stability
Source: Vision (Basel). 2019 Sep 24;3(4):49. doi: 10.3390/vision3040049 (PMC6969937; doi:10.3390/vision3040049)

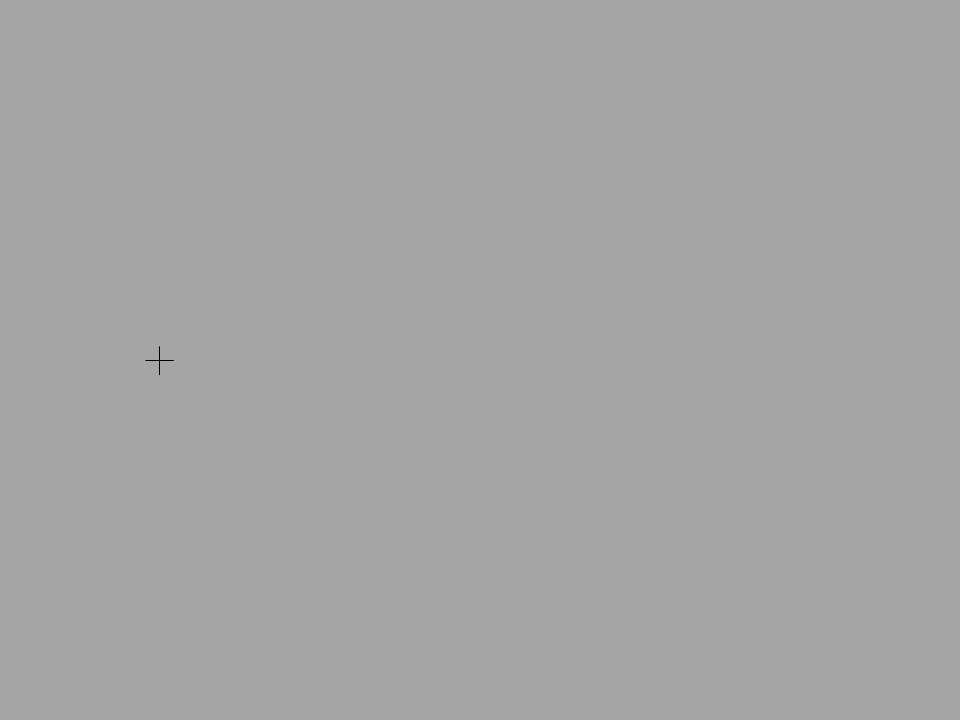

Supplement: Supplementary file 1 [file vision-03-00049-s001.zip › BornSupplement/MovieS1_Control.gif]

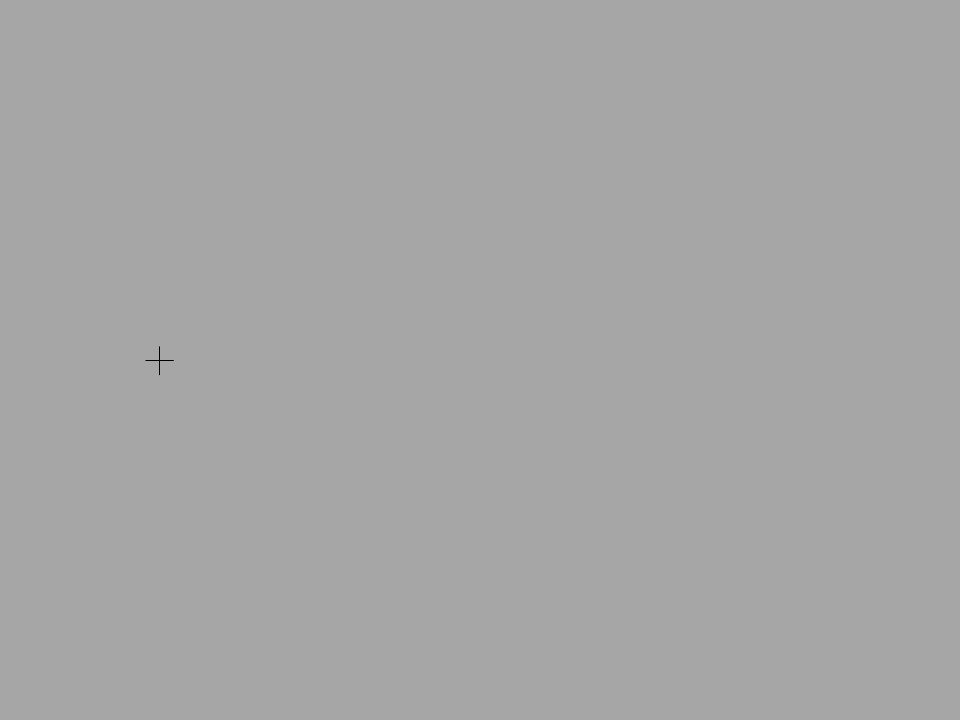

Supplement: Supplementary file 1 [file vision-03-00049-s001.zip › BornSupplement/MovieS2_Mask.gif]

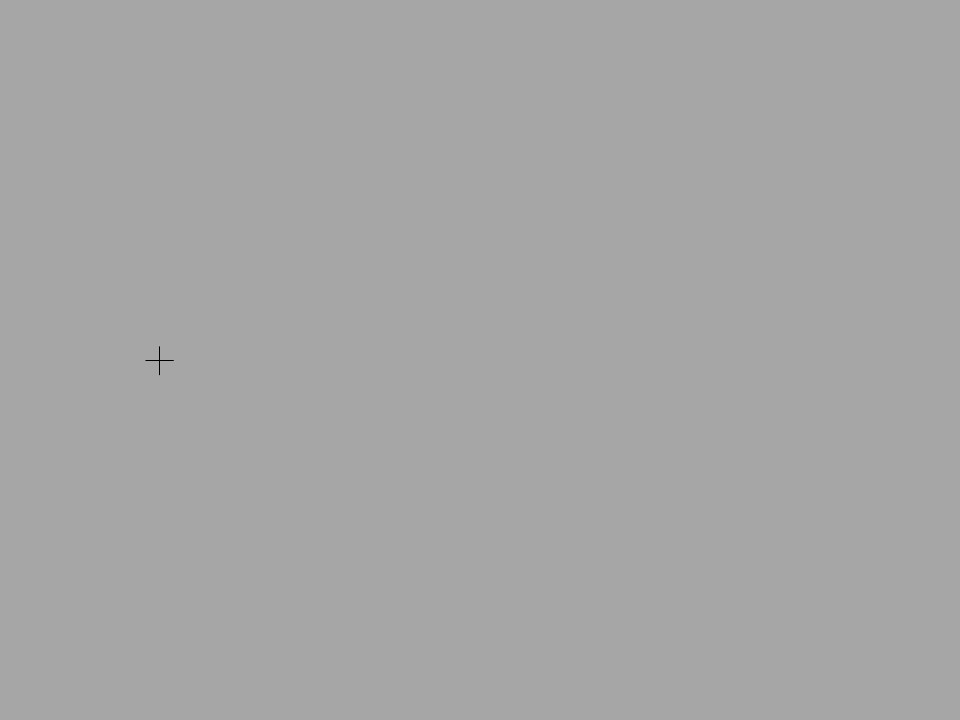

Supplement: Supplementary file 1 [file vision-03-00049-s001.zip › BornSupplement/MovieS3_BlankMask.gif]

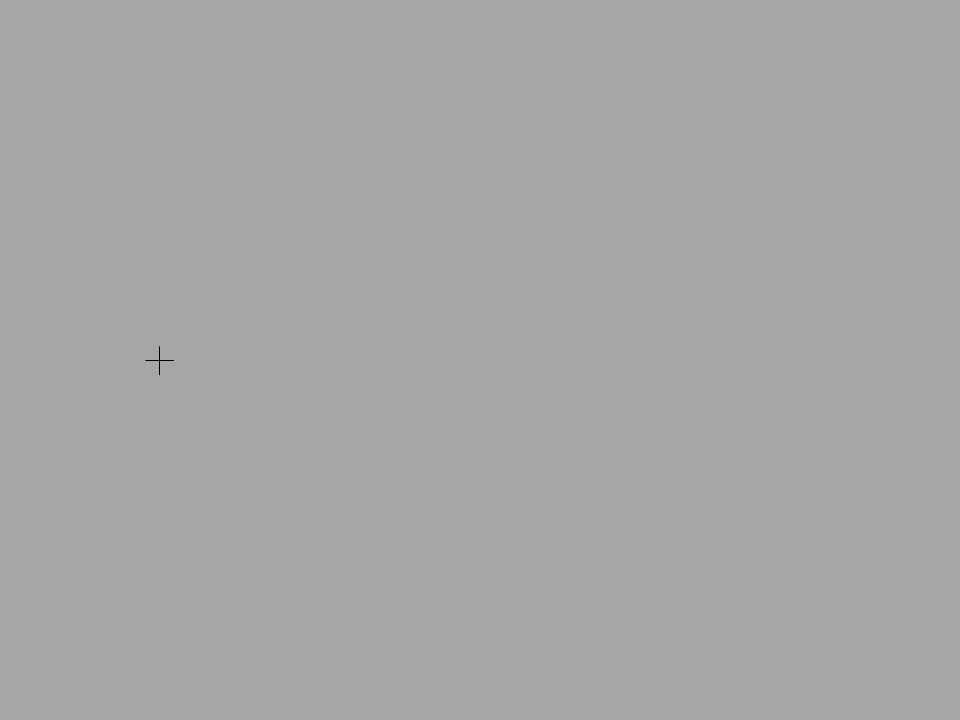

Supplement: Supplementary file 1 [file vision-03-00049-s001.zip › BornSupplement/MovieS4_BlankControl.gif]
